# Development and validation of a quick screening tool for predicting neck pain patients benefiting from spinal manipulation: a machine learning study

**DOI:** 10.1186/s13020-025-01131-z

**Published:** 2025-05-27

**Authors:** Changxiao Han, Guangyi Yang, Haibao Wen, Minrui Fu, Bochen Peng, Bo Xu, Xunlu Yin, Ping Wang, Liguo Zhu, Minshan Feng

**Affiliations:** 1 Wangjing Hospital of China Academy of Chinese Medical Sciences, Beijing, 100102 China; 2 Beijing Key Laboratory of Digital Intelligence Traditional Chinese Medicine for Preventing and Treating Degenerative Bone and Joint Diseases, Beijing, 100102 China; 3 Beijing University of Chinese Medicine, Beijing, 100102 China; 4 First Teaching Hospitnl of Tianjin University of Traditional Chinese Medicine, Tianjin, 300381 China

**Keywords:** Spinal manipulation, Neck pain, Machine learning, Prediction model, SHAP

## Abstract

**Background:**

Neck pain (NP) ranks among the leading causes of years lived with disability worldwide. While spinal manipulation is a common physical therapy intervention for NP, its variable patient responses and inherent risks necessitate careful patient selection. This study aims to develop and validate a machine learning-based prediction model to identify NP patients most likely to benefit from spinal manipulation.

**Methods:**

This multicenter study analyzed 623 NP patients in a retrospective cohort and 319 patients from a separate hospital for external validation, with data collected between May 2020 and November 2024. Treatment success was defined as achieving ≥ 50% reduction in Numerical Rating Scale (NRS) and ≥ 30% reduction in Neck Disability Index (NDI) after two weeks of spinal manipulation. We compared data imputation methods through density plots, and conducted δ-adjusted sensitivity analysis. Then employed both Boruta algorithm and LASSO regression to select relevant predictors from 40 initial features, and four feature subsets (Boruta-selected, LASSO-selected, intersection, and union) were evaluated to determine the optimal combination. Nine machine learning algorithms were tested using internal validation (70% training, 30% testing) and external validation. Performance metrics included Area Under the Receiver Operating Characteristic Curve (AUC), accuracy, F1-score, sensitivity, specificity, and predictive values. The SHAP framework enhanced model interpretability. Youden’s Index was applied to determine the optimal predictive probability threshold for clinical decision support, and a web-based application was developed for clinical implementation.

**Results:**

The combined LASSO and Boruta algorithms identified nine optimal predictors, with the union feature set achieving superior performance. Among the algorithms tested, the Multilayer Perceptron (MLP) model demonstrated optimal performance with an AUC of 0.823 (95% CI 0.750, 0.874) in the test set, showing consistency between training (AUC = 0.829) and test performance. External validation confirmed robust performance (AUC: 0.824, accuracy: 0.765, F1 score: 0.76) with satisfactory calibration (Brier score = 0.170). SHAP analysis highlighted the significant predictive value of clinical measurements and patient characteristics. Based on Youden’s Index, the optimal predictive probability threshold was 0.603, yielding a sensitivity of 0.762 and specificity of 0.802. The model was implemented as a web-based application providing real-time probability calculations and interactive SHAP force plots.

**Conclusion:**

Our machine learning model demonstrates robust performance in identifying suitable candidates for spinal manipulation among neck pain patients, offering clinicians an evidence-based practical tool to optimize patient selection and potentially improve treatment outcomes.

**Supplementary Information:**

The online version contains supplementary material available at 10.1186/s13020-025-01131-z.

## Introduction

Neck pain (NP) represents one of the leading causes of years lived with disability worldwide, according to the Global Burden of Disease Study [1]. Most people can expect to have some degree of neck pain within their lifetime. The condition frequently presents with comorbid disorders, including cervicogenic headache and radiating pain into the upper extremities, resulting in significant functional limitations and disability [2]. Globally, the annual incidence of NP ranged between 10.4% and 21.3%, with higher rates observed in females and older populations [3]. In China, the lifetime prevalence of NP has reached 8.1% to 19.1%, demonstrating an upward trend in recent years and generating healthcare expenditures amounting to hundreds of millions of yuan [4, 5].

Over 50% of patients with neck pain seek physical therapy, accounting for approximately 25% of all physical therapy consultations [6, 7]. Among various treatment approaches, spinal manipulation (SMT) has emerged as one of the most commonly recommended physical therapy interventions for neck pain management [8]. This technique involves high-velocity, low-amplitude manipulations applied to the cervical spine and surrounding soft tissues to reduce pain, enhance mobility, and modulate neuromuscular responses [9, 10]. Despite strong supporting evidence [11–15], physical therapists may be reluctant to employ spinal manipulation due to inherent procedural risks [16–18]. Moreover, treatment outcomes demonstrate considerable prognostic and response variability across populations, suggesting that positive results from manual therapy may be more closely associated with individual patient responsiveness rather than specific pathological conditions [19]. This variability and unpredictability in treatment response necessitates therapists to evaluate whether potential benefits outweigh inherent risks, highlighting the importance of identifying appropriate candidates for manual therapy intervention.

To address this clinical challenge, several studies have developed Clinical Prediction Rules (CPRs) [20–25]. For example, Puentedura et al. [21] developed a clinical prediction rule to identify patients with neck pain who would likely benefit from manipulation. They identified four key predictors (symptom duration less than 38 days, positive expectation of manipulation, side-to-side difference in cervical rotation range of motion ≥ 10°, and pain with posteroanterior spring testing of the middle cervical spine) and the presence of 3 or more predictors increased the probability of successful treatment from 39 to 90% (positive likelihood ratio = 13.5). Similarly, Cleland et al [24]. established a clinical prediction rule through a prospective cohort study, identifying six predictor variables, with the presence of ≥ 3 variables increasing the success rate from 54 to 86% (positive likelihood ratio = 5.5). These studies generally developed CPRs based on favorable outcomes using patient demographics, medical history, clinical assessments, and patient-reported measures.

However, these efforts have predominantly relied on traditional statistical methods, employing univariate or multivariate analyses that often overlook potential covariance or nonlinear relationships between variables. Such approaches have limited effectiveness in addressing nonlinear problems inherent in clinical prediction. Moreover, these methods typically yield results in the form of specific indicator ranges, which not only have limited clinical applicability but may also fail to identify potentially responsive patients who fall outside these predetermined ranges. Importantly, these studies were constrained by limited sample sizes (Puentedura et al. [21]: n = 82, Cleland et al. [24]: n = 78, Flynn et al [25]: n = 71, Iverson et al. [23]: n = 50, Deyle et al. [22]: n = 101), potentially affecting their generalizability and robustness.

Machine learning (ML) offers unique advantages in addressing these limitations by capturing complex, nonlinear interactions between variables and outcomes [26]. Unlike traditional statistical methods, ML excels at processing high-dimensional data and complex feature relationships, potentially yielding more accurate and comprehensive predictions [27]. Despite these advantages, to our knowledge, no ML-based studies have specifically focused on predicting SMT benefits in NP patients.

To address this gap, we developed and validated a ML-based prediction model to identify NP patients most likely to benefit from SMT. We implemented the Boruta algorithm and LASSO regression for optimal feature selection, ensuring both accuracy and clinical relevance. Model interpretability was enhanced through the SHapley Additive exPlanations (SHAP) framework, providing transparent insights into prediction mechanisms. To facilitate clinical implementation, we developed a web-based application using Streamlit, making the tool readily accessible to clinicians. This study aimed to create an evidence-based clinical decision support tool for personalizing SMT approaches.

## Method

### Study population

**Fig. 1:**
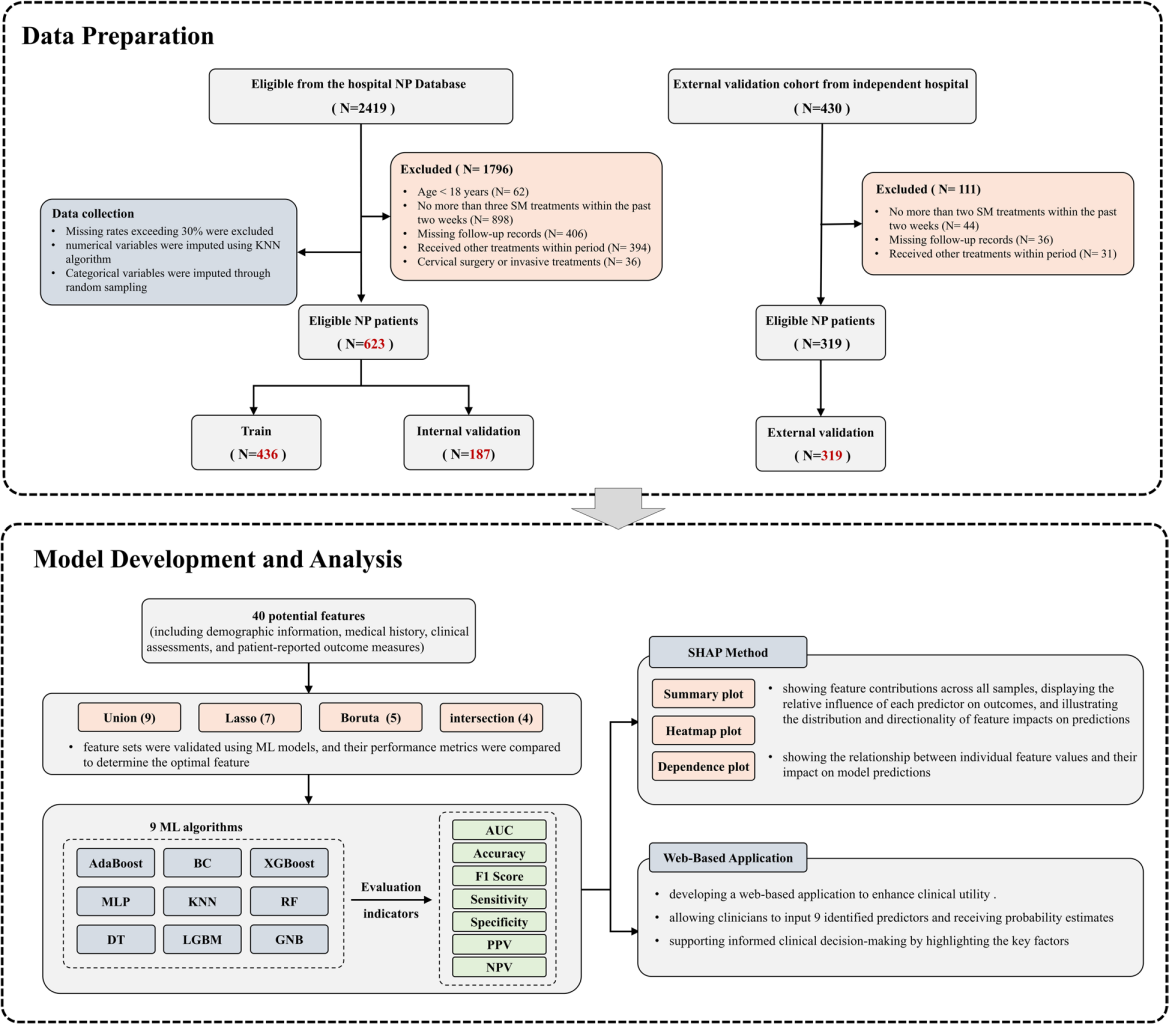
Flow chart of the study design. *NP* neck pain, *ML* machine learning, *SHAP* SHapley Additive Explanation, *AUC* Area Under the Receiver Operating Characteristic Curve, *PPV* positive predictive value, *NPV* negative predictive value, *MLP* multilayer perceptron, *RF* Random Forest, *DT* decision tree, *KNN* k-nearest neighbors, *LGBM* Light Gradient Boosting Machine, *XGBoost* eXtreme Gradient Boosting, *GNB* Gaussian Naive Bayes, *BC* Bagging Classifier, *KNN* K-Nearest Neighbors, *LASSO* Least Absolute Shrinkage and Selection Operator

This is a retrospective cohort study using data sourced from the Wangjing Hospital of China Academy of Chinese Medical Sciences neck pain database (http://www.tcmidc.com:8888/cdc/cs). Data collection occurred between May 2020 and November 2024. The study aimed to develop and validate a machine learning-based predictive model to identify neck pain patients likely to benefit from spinal manipulationtreatment (SMT). Data from the database were anonymized, and no patient consent was required for inclusion in the study. The study was approved by the institutional review board of the Wangjing Hospital, in accordance with the Declaration of Helsinki (WJEC-KT-2023-017-P002) (Fig. 1).

### Eligibility criteria

Participants were eligible if they met the following criteria: age ≥ 18 years, presenting with mechanical neck pain, restricted cervical range of motion, and localized cervical pain symptoms with or without radiating pain to the upper limbs, and having received three SMT within a two-week period. In accordance with strict SMT indications and contraindications, patients were excluded if they presented with medical "red flags" indicating potential non-musculoskeletal pathologies (e.g., tumors, infections, or inflammatory arthritis), history of whiplash injury or acute cervical trauma within the previous six weeks, diagnosed cervical spinal stenosis with symptoms of spinal cord compression, evidence of central nervous system involvement (e.g., hyperreflexia, clonus, or positive Babinski sign), nerve root compression with at least two of the following signs: diminished myotomal strength, altered sensation, or abnormal reflexes, cervical spine surgery or invasive treatments for neck pain within the past year, or contraindications to SM including osteoporosis, ongoing anticoagulation therapy, or severe cardiovascular disease.

### Intervention and outcome definition

All patients underwent a standardized SMT procedure, which typically includes pre-traction and a combination of high-speed, low-amplitude manipulations. Taking the right side as an example (Figure S1), the procedure included the following steps. Preparation: Subjects were seated upright, and the physiotherapist stood behind them. Rotation-Anteflexion Position: The patient’s head was guided to rotate to the right directional limit, then flexed, and finally rotated to the right directional limit again. Preload: The physiotherapist held the patient’s mandible with their forearm and slowly applied upward traction for 3–5 s. Upward-Thrust: Following pretraction, the head was rapidly thrust upward, typically accompanied by an audible “click” sound [28–30].

Each patient received at least three SMT sessions within a two-week period, with outcome assessments conducted two weeks after the first session. Baseline data were collected within 24 h prior to therapy initiation, and follow-up assessments were conducted to evaluate outcome changes. All treatments were performed by licensed physical therapists with over five years of clinical experience, adhering to the above-mentioned standardized protocol.

Outcomes were dichotomized into high-benefit (1) and low-benefit (0) groups. The high-benefit outcome was defined as meeting both of the following criteria at the two-week follow-up after SMT: (1) a reduction of 50% or greater in Numerical Rating Scale (NRS) [31], and (2) a reduction of 30% or greater in their Neck Disability Index (NDI) [32] score from baseline. This dual-criteria definition was specifically chosen to maximize the likelihood that observed improvements were attributable to the intervention rather than natural symptom resolution or other external factors [33]. The combination of these two validated measures also helped minimize potential assessment bias, as it incorporated both patient-reported pain relief (NRS) and functional status change (NDI). All assessments were conducted by independent investigators blinded to treatment allocation to ensure reliable and unbiased data collection for predictive modeling.

### External validation

To assess the generalizability of our prediction model, we conducted external validation using an independent retrospective cohort of neck pain patients from the Tianjin University of Traditional Chinese Medicine Affiliated Hospital collected from January 2022 to December 2024 (ChiCTR2400082667). The inclusion and exclusion criteria were identical to those applied in the derivation cohort, and all patients received the same standardized rotation-traction manipulation protocol as described in the primary study. The validation cohort underwent the same assessment procedures, including collection of demographic information, clinical measurements, and outcome evaluations.

### Model development and analysis

- Step 1: Feature selection

Based on literature review, expert clinical opinions, and retrospective data analysis, we identified 40 potential predictors, including demographic information, medical history, clinical assessments, and patient-reported outcome measures [34–36]. For missing data management, variables with missing rates exceeding 30% were first excluded from the analysis. For the remaining variables, different imputation strategies were applied based on the type of data. To determine the most suitable imputation method, we compared the imputed data distributions to the original data using density plots. For numerical variables, we compared several imputation methods, including Random Forest (RF), Predictive Mean Matching (PMM), mean imputation, Bayesian Linear regression, Least Absolute Shrinkage and Selection Operator (LASSO) regression, K-Nearest Neighbors (KNN), and random sampling. For categorical variables, the methods compared included RF, PMM, weighted PMM, Classification and Regression Trees (CART), and random sampling. Additionally, we performed a sensitivity analysis based on δ adjustments to assess the impact of different imputation methods on the results. For numerical variables, δ values of 25%, 50%, and 100% were applied, and for categorical variables, δ values of -1 and 1 were used, while ensuring that the adjustments remained within reasonable data ranges.

To enhance clinical practicality and feature accessibility, we employed two feature selection methods: the Boruta algorithm and LASSO regression. The Boruta algorithm, a wrapper-based method, was used to identify features with the strongest relevance to the dependent variable, rather than simply optimizing a compact subset for a specific model [37]. Through iterative elimination of low-correlation features, this approach effectively reduced signal noise. For LASSO regression, we determined the optimal features corresponding to λ1se through fivefold cross-validation, ensuring the inclusion of essential predictors while avoiding overfitting [38]. We then evaluated four different feature subsets: those selected by LASSO alone, by Boruta alone, by the intersection of both methods, and by the union of both methods. These feature sets were validated using ML models, and their performance metrics were compared to determine the optimal feature subset for our final model.

- Step 2: Model development

The dataset was split into training (70%) and test (30%) sets. We evaluated nine ML algorithms: Adaptive Boosting (AdaBoost), Multilayer Perceptron (MLP), Decision Tree (DT), KNN, Light Gradient Boosting Machine (LGBM), eXtreme Gradient Boosting (XGBoost), Random Forest (RF), Gaussian Naive Bayes (GNB), and Bagging Classifier (BC). Hyperparameter optimization was performed using grid search with specific parameter grids defined for each algorithm. Model performance was assessed using Area Under the Receiver Operating Characteristic Curve (AUC), accuracy, F1-score, sensitivity, specificity, positive predictive value (PPV), and negative predictive value (NPV). To validate the robustness of the optimal model and mitigate potential overfitting, tenfold cross-validation was applied and external validation was conducted using an independent cohort.

- Step 3: Model interpretation

To address the “black-box” nature of ML models, the SHAP method was applied to rank feature importance and interpret model predictions [39]. SHAP provided both global and local explanations: global explanations illustrated the overall importance of each feature across the dataset, while local explanations demonstrated specific predictions for individual cases based on their unique input data.

- Step 4: Determination of decision thresholds and application development

To optimize model utility in clinical decision-making, we employed Youden’s Index to determine the optimal cutoff value for predicted probability. Youden's Index, defined as sensitivity plus specificity minus one (J = sensitivity + specificity—1), identifies the threshold where sensitivity and specificity achieve optimal balance. This method maximizes identification of true beneficiaries while minimizing misclassification of non-beneficiaries. We calculated predicted probabilities for each patient in the test set using the trained MLP model, evaluated performance metrics across probability thresholds (0.01 to 0.99, incremented by 0.01), and selected the threshold yielding the maximum Youden’s Index as the optimal cutoff. This cutoff value provides clinicians with clear prescription guidance, recommending spinal manipulation therapy when a patient’s predicted probability of benefit exceeds this threshold. To enhance the clinical utility of the final prediction model, it was implemented into a web-based application using the Python-based Streamlit framework. This application allows clinicians to input values for the model’s selected features, returning the probability of the predicted outcome alongside a visual force plot to provide interpretative support for individual cases.

### Statistical analysis

Continuous variables were summarized as mean ± standard deviation (SD) for normally distributed data or median (interquartile range, IQR) for skewed distributions, while categorical variables were described as frequencies and percentages. Statistical comparisons were made using Chi-square tests for categorical variables, independent sample t-tests for normally distributed continuous variables, and Mann–Whitney U tests for skewed continuous variables. The prediction model was built implementing 10 ML algorithms. Data analyses were performed using Python (v3.9.13) and R (v4.4.2), and a two-tailed P-value < 0.05 was considered statistically significant.

## Results

### Demographics and baseline characteristics

A total of 623 patients were included in the final analysis, of whom 335 (53.8%) were classified as high-benefit and 288 (46.2%) as low-benefit following SMT intervention. 40 features were included (Table 1). The specific descriptions of the features (Table S1) and the missing values for each feature (Table S2) were provided in Supplementary. The feature selection process for the analysis showed no significant multicollinearity, with the highest correlation between any two variables being 0.68, well below the 0.8 threshold typically associated with problematic collinearity. Demographic analysis revealed that patients in the high-benefit group were significantly younger (40.92 ± 10.07 vs. 43.58 ± 9.91 years, p = 0.001) and presented with substantially shorter symptom duration (22.06 ± 26.25 vs. 58.31 ± 64.91 months, p < 0.001). The prevalence of previous NP episodes was notably lower in the high-benefit group (43.3% vs. 54.9%, p = 0.006). Gender distribution and BMI showed no significant differences between groups (p = 0.22 and p = 0.986, respectively).

Clinical examination findings demonstrated distinct patterns between groups. The high-benefit group exhibited more restricted baseline cervical range of motion across multiple parameters: flexion (36.66 ± 8.48° vs. 40.70 ± 8.24°, p < 0.001), extension (32.12 ± 7.62° vs. 35.12 ± 7.81°, p < 0.001), and rotation (56.22 ± 9.29° vs. 60.39 ± 9.31°, p < 0.001). Physical assessment revealed a higher prevalence of extensive muscle tension in the high-benefit group (29.6% vs. 12.5%, p < 0.001), along with more frequent pain exacerbation during cervical movements: flexion (67.2% vs. 48.6%, p < 0.001), extension (64.5% vs. 37.5%, p < 0.001), and rotation (56.7% vs. 39.6%, p < 0.001). No significant differences were observed in activities of daily living parameters, including personal care, lifting heavy objects, reading, work capacity, and recreational activities (all p > 0.05). The comparison of characteristics among the training, internal validation, and external validation sets is shown in Supplementary (Table S3).

**Table 1:** Baseline Characteristics and Clinical Assessment Results of NP Patients in the Benefiting and Non-Benefiting Groups

Features	Low-benefit (n = 288)	High-benefit (n = 335)	*P* value
Age (mean (SD))	43.58 (9.91)	40.92 (10.07)	0.001*
Gender (%)			
Male	200 (69.4)	216 (64.5)	0.220
Female	88 (30.6)	119 (35.5)	
BMI (mean (SD))	23.83 (1.78)	23.83 (1.97)	0.986
Symptom duration (mean (SD))	58.49 (64.63)	22.78 (26.96)	< 0.001*
Prior history of NP (%)			
Yes	159 (55.2)	147 (43.9)	0.006*
No	129 (44.8)	188 (56.1)	
Pain laterality (%)			
Yes	145 (50.3)	157 (46.9)	0.560
No	143 (49.7)	178 (53.1)	
Distal to upper back (%)			
Yes	156 (54.2)	163 (48.7)	0.593
No	132 (45.8)	172 (51.3)	
Distal to shoulder (%)			
Yes	141 (49.0)	169 (50.4)	0.510
No	147 (51.0)	166 (49.6)	
Distal to occiput (%)			
Yes	91 (31.6)	91 (27.2)	0.446
No	197 (68.4)	244 (72.8)	
Radiating pain to upper limb (%)			
Yes	74 (25.7)	87 (26.0)	< 0.001*
No	214 (74.3)	248 (74)	
Pain intensity (mean (SD))	1.99 (1.05)	2.09 (1.11)	0.244
Personal care (mean (SD))	1.86 (1.57)	1.75 (1.53)	0.352
Lifting heavy objects (mean (SD))	1.72 (1.50)	1.68 (1.42)	0.755
Reading (mean (SD))	1.99 (1.26)	1.85 (1.20)	0.168
Headache (mean (SD))	1.31 (1.61)	1.21 (1.55)	0.453
Concentration (mean (SD))	1.72 (1.10)	1.81 (1.14)	0.298
Work (mean (SD))	2.04 (1.11)	1.93 (1.19)	0.245
Sleep (mean (SD))	1.96 (1.17)	1.90 (1.17)	0.544
Driving (mean (SD))	1.93 (1.14)	1.99 (1.20)	0.527
Recreational activities (mean (SD))	1.70 (1.47)	1.72 (1.50)	0.864
NDI (mean (SD))	36.43 (7.93)	35.88 (8.26)	0.400
NRS (mean (SD))	3.92 (1.77)	4.10 (1.85)	0.222
Flexion (mean (SD))	40.70 (8.24)	36.66 (8.48)	< 0.001*
Extension (mean (SD))	35.12 (7.81)	32.12 (7.62)	< 0.001*
Lateral flexion (mean (SD))	32.42 (5.64)	31.41 (5.73)	0.028*
Rotation (mean (SD))	60.39 (9.31)	56.22 (9.29)	< 0.001*
Forward head posture (%)			
Yes	45 (15.6)	40 (11.9)	0.223
No	243 (84.4)	295 (88.1)	
Shoulder protraction (%)			
Yes	44 (15.3)	56 (16.7)	0.705
No	244 (84.7)	279 (83.3)	
Shoulders not level (%)			
Yes	29 (10.1)	35 (10.4)	0.982
No	259 (89.9)	300 (89.6)	
Thoracic spine kyphosis (%)			
Yes	73 (25.3)	78 (23.3)	0.613
No	215 (74.7)	257 (76.7)	
DCFET (mean (SD))	20.21 (8.12)	20.83 (7.24)	0.319
Spurling test (%)			
Yes	46 (16.0)	57 (17.0)	0.809
No	242 (84.0)	278 (83.0)	
Spring test pain (%)			
Normal	54 (18.8)	30 (9.0)	< 0.001*
Single segment tenderness	97 (33.7)	109 (32.5)	
2–3 segments tenderness	127 (44.1)	150 (44.8)	
Extensive tenderness	10 (3.5)	46 (13.7)	
Spring test hypomobility (%)			
Normal	59 (20.5)	63 (18.8)	0.954
Single segment tenderness	91 (31.6)	107 (31.9)	
2–3 segments tenderness	82 (28.5)	96 (28.7)	
Extensive tenderness	56 (19.4)	69 (20.6)	
Spring test hypermobility (%)			
Normal	152 (52.8)	177 (52.8)	0.071
Single segment tenderness	57 (19.8)	63 (18.8)	
2–3 segments tenderness	35 (12.2)	61 (18.2)	
Extensive tenderness	44 (15.3)	34 (10.1)	
Muscle tightness (%)			
Normal	71 (24.7)	33 (9.9)	< 0.001*
Single muscle tension	87 (30.2)	71 (21.2)	
2–3 muscles or muscle groups tension	94 (32.6)	131 (39.1)	
Extensive muscle tension	36 (12.5)	100 (29.9)	
Exacerbation on flexion (%)			
Yes	140 (48.6)	225 (67.2)	< 0.001*
No	148 (51.4)	110 (32.8)	
Exacerbation on extension (%)			
Yes	108 (37.5)	216 (64.5)	< 0.001*
No	180 (62.5)	119 (35.5)	
Exacerbation on lateral flexion (%)			
Yes	140 (48.6)	197 (58.8)	0.014*
No	148 (51.4)	138 (41.2)	
Exacerbation on rotation (%)			
Yes	114 (39.6)	190 (56.7)	< 0.001*
No	174 (60.4)	145 (43.3)	

Continuous variables are presented as mean (standard deviation). Categorical variables are presented as number (percentage)

*BMI* Body mass index, *DCFET* Deep cervical flexor endurance Test, *NRS* Numeric pain rating scale, *NDI* Neck disability index

### Feature selection

For numerical variables, the imputed data using KNN were found to closely match the original data distribution, while for categorical variables, imputation through random sampling was shown to result in distributions that were consistent with the original data (Figure S2). Therefore, numerical variables were imputed using the KNN algorithm, which fills missing values by calculating similarity between samples, while categorical variables were imputed through random sampling based on their existing probability distributions. The results from the δ-adjusted sensitivity analysis showed that the estimated results were very similar to the original estimates, with consistent directions and no changes in the statistical conclusions (Table S4, Table S5). This confirms that the inferences based on the missing data assumptions are robust and that the imputation methods used in this study did not introduce significant biases in the results.

**Fig. 2:**
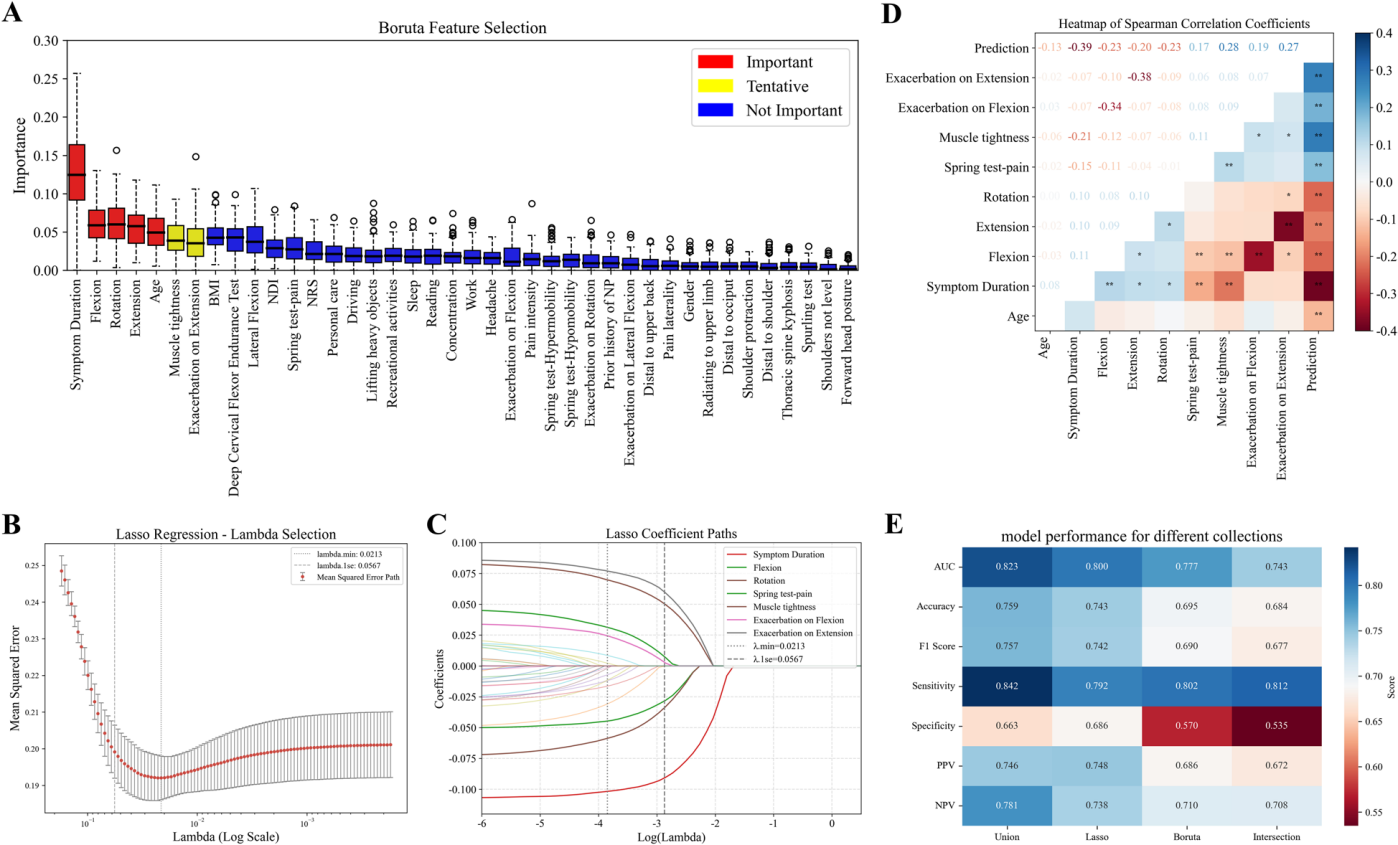
Feature selection and performance evaluation for predicting the benefit of spinal manipulation in neck pain patients. **A** Boruta algorithm feature selection results, with selected features (blue) and rejected features (red) ranked by their Z-scores. **B** Lasso regression cross-validation process to determine the optimal regularization parameter (λ). The Mean Squared Error (MSE) path is shown for different λ values, with λ_min (most regularized) and λ_1se (simpler model) highlighted. **C** Coefficient paths of all 40 features as a function of log-transformed λ, showing the shrinkage process and the selection of important predictors. **D** The final selected features Heatmap of Spearman Correlation Coefficients. **E** Performance comparison of four feature sets (LASSO, Boruta, their intersection, and their union)

To identify the optimal predictors for SM benefit in NP patients, we employed a dual-method feature selection approach combining LASSO regression and the Boruta algorithm, followed by comprehensive performance evaluation of different feature subsets (Table S6). The LASSO regression cross-validation process (Fig. 2B) identified an optimal regularization parameter λ1se of 0.057, representing the largest λ within one standard error of the minimum mean squared error (λmin = 0.021). The coefficient paths (Fig. 2C) illustrate the sequential variable elimination process across different λ values, demonstrating the stability and importance of selected features. The Boruta algorithm, as shown in Fig. 2A, evaluated feature importance through comparison with randomly permuted shadow features. Features were classified as selected (blue) or rejected (red) based on their relative importance scores (Z-scores).

Performance evaluation across the four feature subsets revealed that the union of LASSO and Boruta features achieved superior performance across most metrics, with an AUC of 0.823, accuracy of 0.759, F1 score of 0.757. The LASSO-only subset demonstrated comparable but slightly lower performance (AUC: 0.800, accuracy: 0.743, F1 score: 0.742). In contrast, both the Boruta-only and intersection subsets showed reduced performance across all metrics, with the intersection subset exhibiting particularly diminished effectiveness (AUC: 0.743, accuracy: 0.684, F1 score: 0.677). This underperformance of the intersection subset can be attributed to its limited feature count (only three features), which, while important by both algorithms' standards, was insufficient to capture the complex patterns within our dataset. Based on these comprehensive evaluations, the union of LASSO and Boruta-selected features was determined to be the optimal feature set for our predictive model. This combined approach leverages the strengths of both selection methods: LASSO’s ability to handle multicollinearity and select features with strong direct impact on the target variable, alongside Boruta’s capacity to identify all relevant features including those with complex nonlinear relationships. The superior performance of the union feature set suggests that both methods identified complementary predictors important for SM benefit prediction in NP patients. Additionally, our assessment of potential multicollinearity using Spearman correlation analysis confirmed that no problematic correlations existed between the selected features, with the highest correlation coefficient being 0.68, well below the conventional 0.8 threshold for concerning collinearity (Fig. 2D, Figure S3).

### Model development and performance comparison

**Fig. 3:**
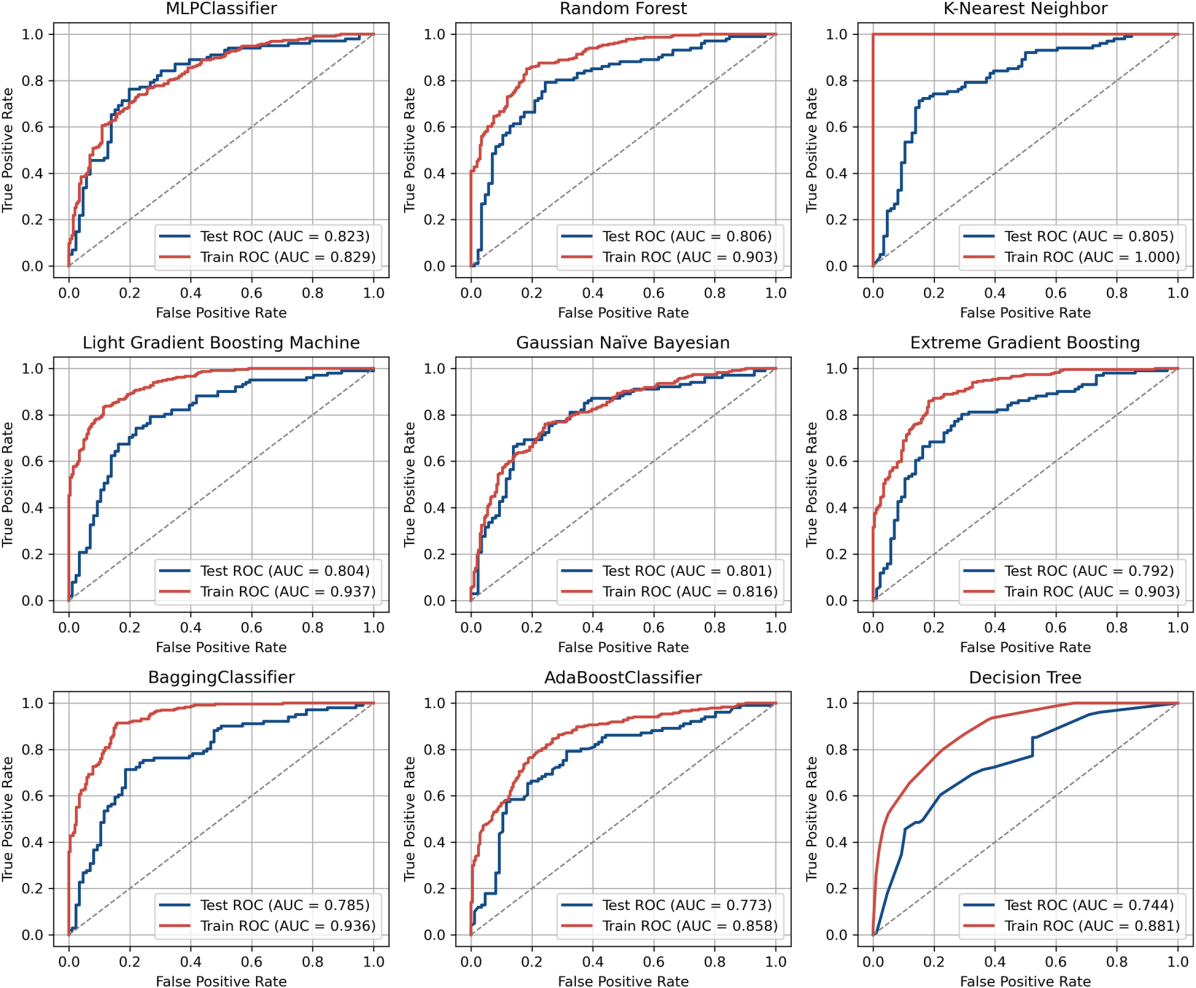
Model performance and calibration for predicting benefit from MT in NP patients. Illustrates the ROC curves of 9 ML models, with AUC values calculated for both training and validation datasets

**Fig. 4:**
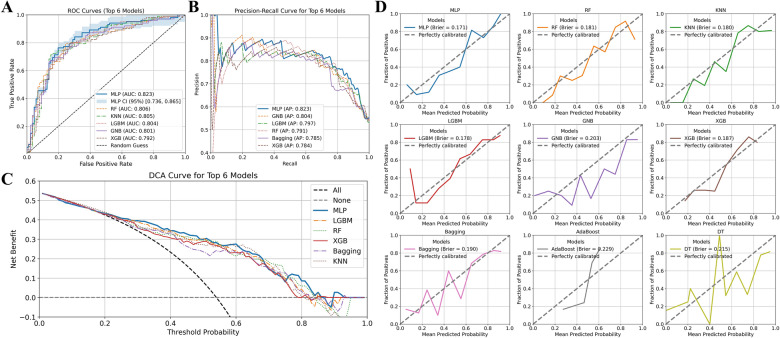
Performance evaluation and clinical utility of nine machine learning models in predicting benefit from spinal manipulation in neck pain patients. **A** ROC curves for the top six models, with AUC values reported for the validation set; **B** PR curves for the top six models; **C** DCA for the top six models. **D** shows the calibration curves for the 9 models, with Brier scores indicating the accuracy of probability estimation

Based on the identified 9 features, models were constructed using 10 ML algorithms. Among them, the MLP demonstrated the best performance with a test AUC of 0.823 (95% CI: 0.750, 0.874), followed by RF with an AUC of 0.806 (Fig. 4A). Furthermore, MLP exhibited minimal discrepancy between the training and test AUC (train AUC = 0.829), indicating excellent generalization capability. In contrast, KNN showed some overfitting. LGBM, XGB, Bagging and DT also demonstrated varying degrees of overfitting (Fig. 3).

In the Precision-Recall (PR) curve analysis, MLP continued to show the best performance, achieving the highest average precision (AP = 0.823), followed by GNB (AP = 0.804) and LGBM (AP = 0.797) (Fig. 4B). Decision Curve Analysis (DCA) was conducted to assess the clinical utility of the models across different threshold probabilities. The results indicated that MLP and LGBM consistently provided the highest net benefit, suggesting their superior practical utility in clinical decision-making (Fig. 4C). Calibration curves were used to evaluate the reliability of predicted probabilities. Both MLP and RF demonstrated calibration curves closely aligned with the ideal diagonal, indicating accurate probability estimation, with Brier scores of 0.171 and 0.181, respectively, reflecting the best calibration performance (Fig. 4D). Overall, the MLP model emerged as the optimal choice, achieving the best results across multiple evaluation metrics (Table 2).

**Table 2:** Predictive Performance of 9 Machine Learning Models for predicting Neck Pain Patients Benefiting from Spinal Manipulation

Model	AUC (95%CI)	Accuracy	F1 score	Sensitivity	Specificity	PPV	NPV
MLP	0.823 (0.742, 0.903)	0.759	0.757	0.842	0.663	0.746	0.781
RF	0.806 (0.724, 0.887)	0.754	0.753	0.802	0.698	0.757	0.75
KNN	0.805 (0.718, 0.891)	0.733	0.731	0.792	0.663	0.734	0.731
LGBM	0.804 (0.715, 0.892)	0.765	0.764	0.792	0.733	0.777	0.75
GNB	0.801 (0.720, 0.882)	0.711	0.699	0.881	0.512	0.679	0.786
XGB	0.792 (0.707, 0.877)	0.749	0.747	0.812	0.674	0.745	0.753
BC	0.785 (0.705, 0.864)	0.695	0.692	0.782	0.593	0.693	0.699
AdaBoost	0.773 (0.699, 0.847)	0.722	0.715	0.851	0.57	0.699	0.766
DT	0.744 (0.666, 0.822)	0.636	0.628	0.772	0.477	0.634	0.641

*AUC* Area Under the Receiver Operating Characteristic Curve, *PPV* Positive predictive value, *NPV* Negative predictive value, *MLP* Multilayer perceptron, *RF* Random Forest, *DT* Decision tree, *KNN* k-Nearest neighbors, *LGBM* Light Gradient Boosting Machine, *XGBoost* eXtreme Gradient Boosting, *GNB* Gaussian Naive Bayes, *BC* Bagging Classifier, *KNN* K-nearest neighbors

MLP Model learning curve analysis (Figure S4A) shows that as sample size increases, training and validation scores gradually converge to stable values, with a final difference stabilizing at approximately 0.03, below the conventional overfitting threshold (0.05), indicating good generalization capacity. tenfold cross-validation analysis (Figure S4B-C) demonstrates stable model performance across different data subsets, with a mean validation AUC of 0.805, standard deviation of only 0.036, and coefficient of variation of 4.47%. The average difference between training and validation AUC is merely 0.019, with three folds even showing negative values (validation performance exceeding training performance), strongly confirming the absence of significant overfitting.

### External validation of the final model

The MLP model demonstrated robust generalizability in the external validation cohort. In external validation, the model achieved an AUC of 0.824, which is comparable to the internal validation performance (AUC = 0.823). The model maintained strong discriminative ability with Accuracy of 0.765, F1 score of 0.76. The precision-recall curve in the external validation cohort yielded an average precision of 0.770, demonstrating consistent performance in identifying patients likely to benefit from SM. Model calibration in the external cohort remained satisfactory, with a Brier score of 0.170, indicating reliable probability estimates. The detailed results of the external validation can be found in the supplementary file (Figure S5). The calibration curve showed good alignment with the ideal diagonal, suggesting accurate prediction of treatment benefit probabilities across different risk levels. Decision curve analysis in the external validation cohort confirmed the model’s clinical utility, with positive net benefit observed across the clinically relevant threshold probability. The model's consistent performance across both internal and external validation cohorts supports its reliability as a clinical decision-support tool for predicting SM outcomes in NP patients.

### Model explanation

**Fig. 5:**
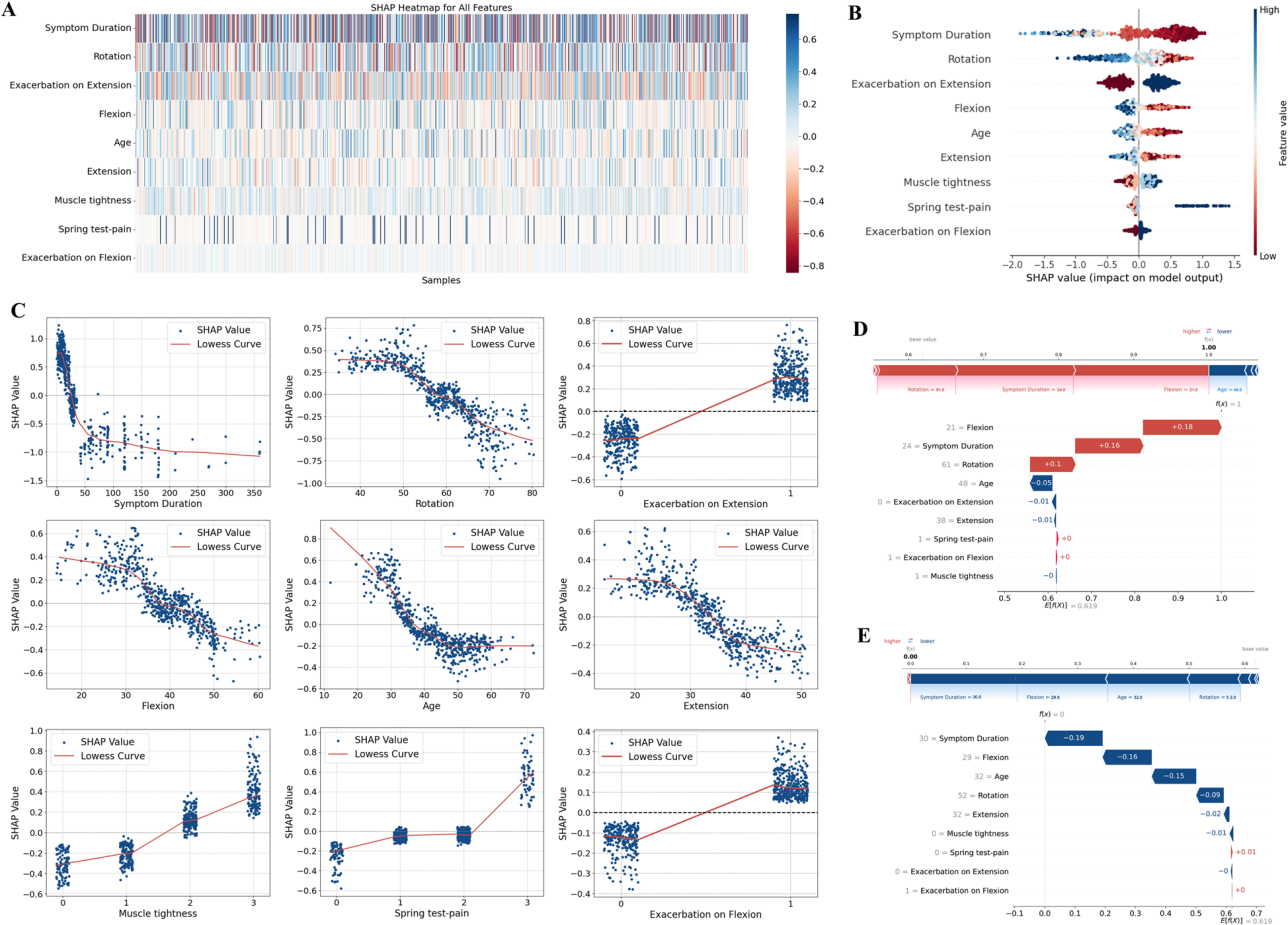
Model interpretation using SHAP analysis for predicting treatment outcomes in neck pain patients. **A** SHAP heatmap showing feature contributions across all samples, with color intensity indicating the magnitude and direction of feature effects (red = positive impact, blue = negative impact). **B** SHAP summary plot for key predictors, illustrating the distribution and directionality of feature impacts on predictions, where red dots indicate increased likelihood and blue dots indicate decreased likelihood of high benefit. **C** SHAP dependence plots showing the relationship between individual feature values and their impact on model predictions, with each point representing a patient and trend lines indicating the overall pattern of feature influence. **D** SHAP force and waterfall plots for a high-benefit patient. **E** SHAP force and waterfall plots for a low-benefit patient

To enhance the interpretability of the model’s predictions, we employed the SHAP method to calculate the contribution of each variable to the final prediction. The global explanation provides insight into the overall feature contributions to the model, while the local explanation helps us understand how specific individual predictions are formed. According to the SHAP feature importance ranking, the most influential features are as follows: Symptom Duration, Rotation, Exacerbation on Extension, Flexion, Age, Extension, Muscle tightness, Spring test-pain and Exacerbation on Flexion. In the global explanation, we utilized the SHAP heatmap (Fig. 5A) and summary plot (Fig. 5B) to illustrate the average contributions of these features, highlighting those that exert the most significant influence on the model’s predictions.

The SHAP dependence plots (Fig. 5C) further demonstrate the relationship between each feature value and the model’s predicted output. These plots aid in understanding how changes in individual features impact the prediction. For instance, in the dependence plot for Symptom Duration, there is a noticeable decline in SHAP values as symptom duration increases, indicating that longer symptom duration is associated with lower predicted outcomes. Conversely, Spring test pain exhibits a positive correlation, where higher values of test pain correspond to higher predicted values. These analyses allow us to gain a deeper understanding of how the model derives its predictions based on each input feature, providing detailed explanations for clinical decision-making and assisting clinicians in interpreting the model’s predictions.

To demonstrate how SHAP outputs contribute to treatment decision-making, we visualized the model's interpretation for both high-benefit and low-benefit groups using SHAP force plots and waterfall plots. Specifically, these plots show the contribution of each feature to the predicted outcome for a particular sample. The impact of each feature is represented by red bars for positive contributions and blue bars for negative contributions, with the lengths of the bars indicating the magnitudes of their respective impacts. For high-benefit patients (Fig. 5D), key features such as symptom duration, flexion and rotation contributed positively to the prediction, while for low-benefit patients (Fig. 5E), features like age, and symptom duration played a negative role in the treatment outcome.

### Thresholds determination and application implementation

**Fig. 6:**
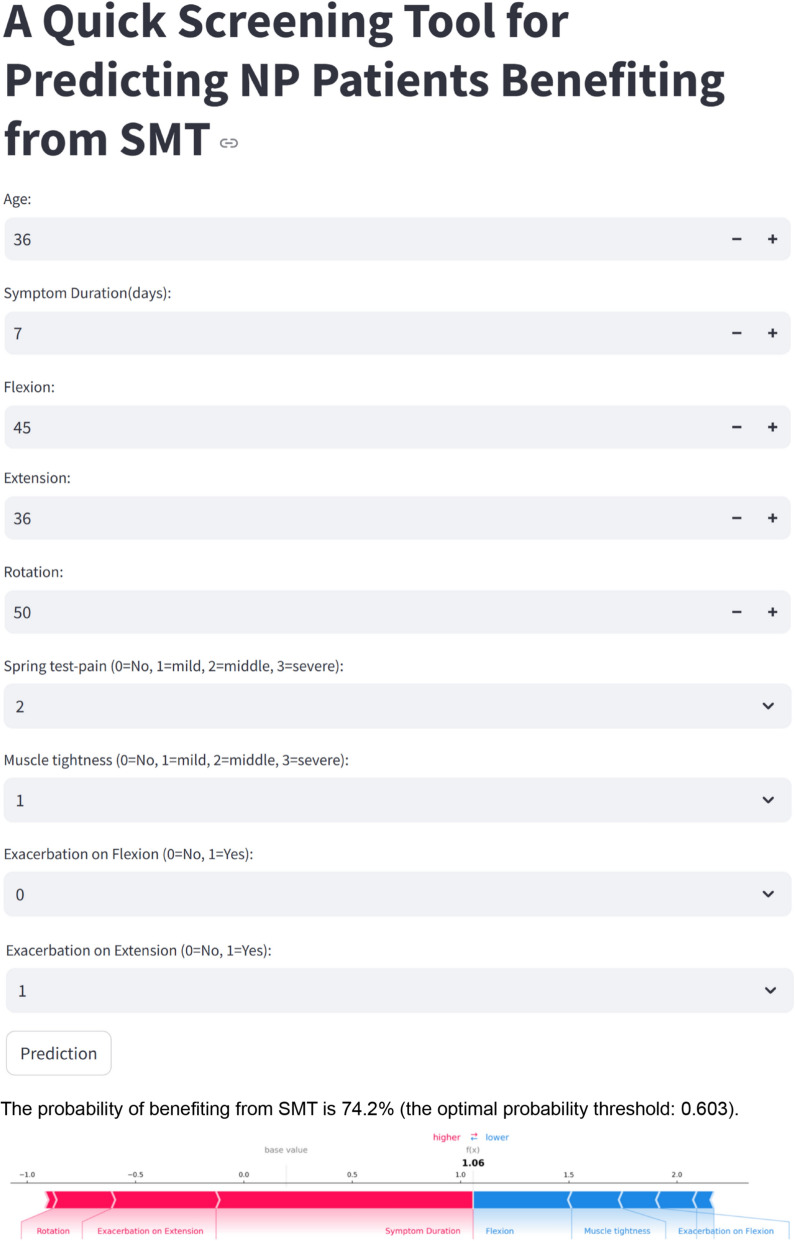
Web-based application incorporating the MLP model for SM benefit. The interface allows clinicians to input values for 9 predictors, and the model provides a probability estimate for SM benefit. Based on the Youden index, the optimal predictive probability threshold has been determined to be 0.603, as indicated on the web. When a patient’s predicted probability of benefit exceeds this threshold, it is strongly recommended to consider spinal manipulation as a primary intervention. Below the prediction, an interactive SHAP force plot highlights individual feature contributions, with red bars indicating factors that increase the predicted probability and blue bars representing factors that decrease it. This visualization aids clinicians in understanding the reasoning behind each prediction, supporting transparent and informed decision-making

Based on Youden’s Index maximization, the optimal predicted probability threshold was determined to be 0.603 (Youden’s Index = 0.565). At this threshold, the model demonstrated balanced performance metrics in the test set: sensitivity of 0.762, specificity of 0.802, accuracy of 0.781, F1 score of 0.790, positive predictive value of 0.819, and negative predictive value of 0.742. These results indicate that when a patient's predicted probability of benefit exceeds 0.603, there is approximately an 81.9% likelihood of significant improvement from spinal manipulation therapy; conversely, when the predicted probability falls below this threshold, there is approximately a 74.2% likelihood of not achieving significant improvement. This cutoff value maintains clinically acceptable sensitivity while providing relatively high specificity, helping reduce unnecessary treatments and potential risks. The cutoff has been integrated into our web application to provide explicit decision support for clinicians (Figure S6). Finally, we developed a web-based application to enhance clinical utility (Fig. 6), requiring approximately 5 min to complete during initial patient assessment. The tool requires input for 9 key predictors identified by our model. (1) Symptom Duration: Obtained through patient interview, recorded in days since symptom onset. (2) Cervical Range of Motion measurements (Rotation, Extension, Flexion): Assessed using standard goniometry during physical examination, recorded in degrees. (3) Age: Obtained from patient demographic information, recorded in years. (4) Spring Test Pain: Assessed by applying posterior-to-anterior pressure to cervical vertebrae, categorized as: 0 = Normal, 1 = Single segment pain, 2 = 2–3 segments pain, 3 = Widespread pain. (5) Muscle Tightness: Evaluated through manual palpation of cervical musculature, categorized as: 0 = Normal, 1 = Single muscle tight, 2 = 2–3 muscles tight, 3 = Widespread tightness. (6) Pain Exacerbation patterns (on Extension and Flexion): Determined by observing symptom response during active cervical movements, recorded as binary outcomes (0 = No, 1 = Yes). The specific operational and acquisition details of key predictors are shown in Figure S7.

All measurements are obtained through standardized clinical assessments routinely performed during initial neck pain evaluations, requiring no specialized equipment beyond standard goniometry. The system provides real-time probability calculations and generates interactive SHAP force plots that display individual feature contributions, with positive and negative predictive factors highlighted in red and blue respectively. These force plots offer clinicians transparent insight into how each patient’s characteristics influence their predicted outcome, thereby supporting informed clinical decision-making by highlighting the key factors driving the prediction in each case. The application is freely accessible at https://SMT-benefit-prediction.streamlit.app, making it a valuable tool for clinical practice.

To demonstrate the practical application of our web application and SHAP interpretation, we present a clinical case as shown in Fig. 6: A 36-year-old patient presents with neck pain of 7 days’ duration. Clinical examination reveals moderate cervical range of motion limitations (flexion 45°, extension 36°, rotation 50°), moderate tenderness on spring testing (level 2—affecting 2–3 segments), and mild muscle tightness (level 1—single muscle tension). Pain is exacerbated with cervical extension but not with flexion movements. When the clinician enters these findings into our application: the predicted probability of benefiting from spinal manipulation treatment (SMT) is 74.2% (exceeding the optimal threshold of 60.3%). The SHAP force plot highlights that key positive contributors to this prediction include the patient's rotation measurements, pain exacerbation with extension, and notably short symptom duration (7 days). Factors that slightly reduce the predicted benefit include the flexion range (which is relatively well-preserved at 45°), mild muscle tightness (rather than more extensive tension patterns), and absence of pain exacerbation with flexion. Based on the 74.2% probability (well above our established threshold of 60.3%), after excluding any contraindications, spinal manipulation would be an appropriate treatment recommendation for this patient. This case illustrates how our model not only provides a probability prediction but also offers transparent reasoning through SHAP visualization, allowing clinicians to identify which specific patient characteristics drive the likelihood of treatment success. This visual representation helps clinicians integrate the algorithmic prediction with their clinical reasoning process.

## Discussion

This study developed and validated a machine learning-based prediction model to identify neck pain patients most likely to benefit from spinal manipulation. To our knowledge, this is the first study to use machine learning to construct such a model. With 623 patients in the development cohort and 319 in the external validation cohort, this represents the largest study to date in this field, substantially exceeding previous studies' sample sizes (typically n < 100). Our research employs advanced machine learning algorithms to fully explore potential covariance and non-linear relationships between variables, achieving excellent predictive performance (AUC: 0.823 in test set, 0.824 in external validation). The model successfully identified nine key predictors that significantly influence treatment outcomes, including symptom duration, cervical range of motion (rotation and extension), pain exacerbation patterns, age, muscle tightness, and spring test pain. Through our comprehensive methodology incorporating multiple machine learning algorithms and robust feature selection techniques, we uncovered complex interactions between these predictors that were not identified in previous studies using conventional statistical methods. Moreover, through SHAP and the developed web application, we effectively highlighted the model's interpretability, acceptability, and clinical utility. Physicians can easily obtain the patient's 9 features and generate interpretable predictions within 5 min during the initial visit. This ensures the model's applicability across various clinical settings and its direct practical value for clinicians.

For feature selection, we applied two widely recognized methods, Lasso and the Boruta algorithm. However, we did not use the intersection of the selected features from both methods as the final feature set, as it only included three features. Although these were deemed important by both algorithms, the limited number of features overly simplified the model, preventing it from capturing all significant patterns and potential relationships in the data. Lasso tends to select features with a stronger impact on the target variable, but due to its sensitivity to feature correlations, it may exclude seemingly unimportant features that, in combination with others, contribute to the model's performance. In contrast, Boruta, based on random forests, compares original features with randomly generated “shadow” features to assess which features are useful in the model. Boruta emphasizes evaluating the importance of features to the model output and is better at capturing complex nonlinear relationships between features. Consequently, Boruta-selected features may include those with strong interactions or nonlinear relationships with other features. Further validation of the four feature sets revealed that the intersection had the poorest predictive performance, while the union showed the best performance. Moreover, no overfitting was observed as the feature set increased, whether in the training set, internal validation set, or external validation set. Therefore, we selected the union as the final feature set.

Among the identified predictive variables, symptom duration and cervical function have been recognized as primary determinants of manual therapy effectiveness, which is consistent with previous findings. In Cleland's study, symptom duration was identified as the strongest predictor among the six variables in the clinical prediction rule, aligning with the present results. Additionally, three of the variables (no aggravation of symptoms with looking up, no symptoms distal to the shoulder, and cervical extension < 30°) were directly related to functional status. In Puentedura et al.’s study, four predictor variables were identified through regression analysis: symptom duration less than 38 days, positive expectation of manipulation, side-to-side difference in cervical rotation range of motion ≥ 10°, and pain with posteroanterior spring testing of the middle cervical spine. Notably, symptom duration remained the primary predictor. This emphasis on both temporal and functional predictors suggests their central role in determining treatment outcomes for cervical spine manipulation. While both studies share similarities in their final predictor variables with the present research. However, in both study, potential predictor variables were first identified through univariate analyse and regression algorithms, followed by receiver-operating-characteristic (ROC) curve analysis to determine optimal cutoff points for continuous variables. Likelihood ratios were then calculated based on 2 × 2 contingency tablesthey. This approach may have overlooked potential relationships and covariance effects between different variables, which could provide additional insights into prediction modeling for treatment outcomes. The findings need further validation through larger sample sizes and randomized controlled trials, but RCTs based on clinical rules have yielded mixed results. Childs et al [40] and Cleland et al [41] further validated clinical prediction rules for spinal manipulation in patients with low back pain, demonstrating positive results. In contrast, Rabin et al [42] and Mintken et al [20] did not fully validate their respective prediction rules. Rabin et al. found that while patients with a positive CPR status experienced less disability with lumbar stabilization exercise, the CPR could not be definitively validated. Similarly, Mintken et al. did not support the prognostic variables for cervicothoracic manipulation in shoulder pain, suggesting the need for further refinement and validation of the prediction rule.

Our machine learning model demonstrated robust performance and strong generalizability in predicting spinal manipulation benefits for neck pain patients. In internal validation, the MLP model achieved an AUC of 0.823 (95% CI 0.750, 0.874) with minimal discrepancy between training and test performance (training AUC = 0.829), indicating excellent model stability. The model maintained comparable performance in external validation (AUC = 0.824), with strong discriminative ability (accuracy = 0.765, F1 score = 0.76) and reliable probability estimation (Brier score = 0.170). The model’s calibration curves showed good alignment with the ideal diagonal in both internal and external validation, suggesting accurate probability estimates across different risk levels. Moreover, decision curve analysis confirmed the model’s clinical utility by demonstrating positive net benefit across clinically relevant threshold probabilities. These findings collectively indicate that our model offers reliable and generalizable predictions for identifying patients likely to benefit from spinal manipulation, making it a valuable tool for clinical decision support.

Several limitations of this study should be acknowledged. First, Although this is a multicenter study, all participating hospitals were in China, potentially limiting the model’s generalizability to populations with different ethnic backgrounds or healthcare systems. Future research should include international or multi-ethnic cohorts to evaluate the model's performance across diverse settings. Second, Our outcome assessment was based on a relatively short follow-up period (2 weeks), which may not fully capture the long-term effects of spinal manipulation or patient prognosis. Given the fluctuating nature of chronic neck pain, future research should incorporate extended monitoring periods (3, 6, and 12 months) to evaluate sustained benefits, potential adverse effects, and recurrence patterns. Third, while we employed multiple validation methods to enhance model robustness, there is currently no standardized approach for calculating sample sizes in machine learning-based prediction models. Fourth, our model only considered pre-treatment variables, while real-world treatment outcomes may also be influenced by factors such as therapist experience and specific manipulation techniques used during treatment. Fifth, regarding our web-based prediction tool, the current implementation exists as a standalone application without integration into existing electronic health records (EHR) systems. Future development will focus on creating standardized APIs to enable seamless EHR integration and reduce duplicate data entry. Finally, although our model includes various clinical parameters, certain psychosocial factors that may influence treatment outcomes were not included due to the retrospective nature of the data, despite being known to be important for manual therapies. Future research should integrate these factors and conduct prospective validation in diverse populations and clinical settings to improve the model's accuracy and broader applicability.

## Conclusion

This study presents the first application of machine learning for predicting spinal manipulation outcomes in neck pain patients. Our model demonstrated robust predictive performance and successfully identified nine key predictors of treatment outcomes. The comprehensive methodology incorporating multiple machine learning algorithms revealed complex predictor interactions previously unidentified using conventional statistical methods. The model’s implementation as a web-based application provides clinicians with an accessible tool for evidence-based decision-making. This approach potentially improving treatment outcomes while considering associated risks. Future research should focus on prospective validation in diverse clinical settings and investigation of long-term outcomes.

## Supplementary Information

Supplementary material 1.

## Data Availability

The datasets used and analyzed during the current study are available from the corresponding author on reasonable request.

## Abbreviations

AdaBoost: Adaptive Boosting
AP: Average precision
AUC: Area Under the Receiver Operating Characteristic Curve
BC: Bagging Classifier
BMI: Body mass index
CI: Confidence Interval
CPR: Clinical prediction rule
DCA: Decision curve analysis
DT: Decision tree
GNB: Gaussian Naive Bayes
IQR: Interquartile range
KNN: K-Nearest neighbors
LASSO: Least Absolute Shrinkage and Selection Operator
LGBM: Light Gradient Boosting Machine
ML: Machine learning
MLP: Multilayer perceptron
NDI: Neck Disability Index
NP: Neck pain
NPV: Negative predictive value
NRS: Numerical Rating Scale
PPV: Positive predictive value
RF: Random Forest
SHAP: SHapley Additive exPlanations
SMT: Spinal manipulation treatment
XGBoost: EXtreme Gradient Boosting
