# Supplementary material for: Development and validation of a quick screening tool for predicting neck pain patients benefiting from spinal manipulation: a machine learning study
Source: Chin Med. 2025 May 27;20:74. doi: 10.1186/s13020-025-01131-z (PMC12107896; doi:10.1186/s13020-025-01131-z)
Supplement: Supplementary file 1 — Supplementary material 1. [file 13020_2025_1131_MOESM1_ESM.docx]

**Table S1 Feature Descriptions.**

| **Features** | **Description** | **Value Range** |
| --- | --- | --- |
| Gender | Gender of the participant | 0 = Male, 1 = Female |
| Age | Age in years | Integer (years) |
| Body Mass Index (BMI) | Body Mass Index, calculated as weight (kg) / height (m²) | Numeric value |
| Profession | Type of occupation | 0 = Sedentary, 1 = Physical labor, 2 = Mixed |
| Symptom Duration | Duration of symptoms in days | Integer (days) |
| Prior history of NP | Prior history of neck pain | 0 = No, 1 = Yes |
| Pain Laterality | Laterality of pain | 0 = Unilateral, 1 = Bilateral |
| Pain Distal to Upper Back | Whether pain radiates to the upper back | 0 = No, 1 = Yes |
| Pain Distal to Shoulder | Whether pain radiates to the shoulder | 0 = No, 1 = Yes |
| Pain Distal to Occiput | Whether pain radiates to the occipital region | 0 = No, 1 = Yes |
| Radiating Pain to Upper Limb | Whether radiating pain is felt in the upper limb | 0 = No, 1 = Yes |
| Morning Stiffness | Presence of morning stiffness | 0 = No, 1 = Yes |
| Nocturnal Pain | Whether pain is aggravated during the night | 0 = No, 1 = Yes |
| Pain intensity | Pain intensity (sub-scores of NDI) | 0-5 (higher score means more severe) |
| Personal Care | Ability to perform personal care (sub-scores of NDI) | 0-5 (higher score means more difficulty) |
| Lifting Heavy Objects | Ability to lift heavy objects (sub-scores of NDI) | 0-5 (higher score means more difficulty) |
| Reading | Ability to read (sub-scores of NDI) | 0-5 (higher score means more difficulty) |
| Headache | Severity of headache (sub-scores of NDI) | 0-5 (higher score means more severe) |
| Concentration | Difficulty in concentration (sub-scores of NDI) | 0-5 (higher score means more difficulty) |
| Work | Difficulty in performing work tasks (sub-scores of NDI) | 0-5 (higher score means more difficulty) |
| Sleep | Sleep quality (sub-scores of NDI) | 0-5 (higher score means worse sleep) |
| Driving | Difficulty in driving (sub-scores of NDI) | 0-5 (higher score means more difficulty) |
| Recreational Activities | Difficulty in performing recreational activities (sub-scores of NDI) | 0-5 (higher score means more difficulty) |
| Neck disability index (NDI) | A questionnaire used to assess how neck pain affects daily activities and quality of life | Numeric value (higher score indicates more disability) |
| Numeric pain rating scale (NRS) | A subjective measure where patients rate their pain on a scale from 0 (no pain) to 10 (worst pain imaginable) | 0-10 (higher score means more pain) |
| Flexion | Flexion range of motion in degrees | Numeric value |
| Extension | Extension range of motion in degrees | Numeric value |
| Lateral Flexion | Lateral flexion range of motion in degrees | Numeric value |
| Rotation | Rotation range of motion in degrees | Numeric value |
| Forward Head Posture | Presence of forward head posture | 0 = No, 1 = Yes |
| Shoulder Protraction | Presence of shoulder protraction | 0 = No, 1 = Yes |
| Shoulders Not Level | Presence of uneven shoulders | 0 = No, 1 = Yes |
| Thoracic Spine Kyphosis | Presence of thoracic spine kyphosis | 0 = No, 1 = Yes |
| Deep Cervical Flexor Endurance Test (DCFET) | Evaluates the endurance of the deep cervical flexor muscles by measuring how long a specific neck posture can be held. | Numeric value (seconds) |
| Spurling Test | Assesses cervical radiculopathy by applying pressure to the neck in a tilted position to reproduce nerve root pain. | 0 = Negative, 1 = Positive |
| Spring Test-pain | Assesses spinal segmental tenderness by applying gentle pressure to specific vertebrae to identify pain response | 0 = Normal, 1 = Single segment pain, 2 = 2-3 segments pain, 3 = Widespread pain |
| Spring Test-Hypomobility | Evaluates restricted movement in specific vertebrae by applying posterior-to-anterior pressure. | 0 = Normal, 1 = Single segment, 2 = 2-3 segments, 3 = Widespread |
| Spring Test-Hypermobility | Assesses excessive movement in specific vertebrae using posterior-to-anterior pressure. | 0 = Normal, 1 = Single segment, 2 = 2-3 segments, 3 = Widespread |
| Muscle Tightness | An increase in tension or stiffness in a muscle, often assessed to determine restrictions in range of motion or muscle dysfunction. | 0 = Normal, 1 = Single muscle tight, 2 = 2-3 muscles tight, 3 = Widespread tightness |
| Exacerbation on Flexion | Exacerbation of symptoms during flexion | 0 = No, 1 = Yes |
| Exacerbation on Extension | Exacerbation of symptoms during extension | 0 = No, 1 = Yes |
| Exacerbation on Lateral Flexion | Exacerbation of symptoms during lateral flexion | 0 = No, 1 = Yes |
| Exacerbation on Rotation | Exacerbation of symptoms during rotation | 0 = No, 1 = Yes |

**Table S2 Missing Values Overview**

| **Feature Name** | **Missing Values** | **Missing Percentage (%)** |
| --- | --- | --- |
| series | 0 | 0 |
| Gender | 0 | 0 |
| Age | 0 | 0 |
| BMI | 75 | 7.96 |
| Profession | 428 | 45.44 |
| Symptom Duration | 18 | 1.91 |
| Prior history of NP | 28 | 2.97 |
| Pain laterality | 0 | 0 |
| Distal to upper back | 0 | 0 |
| Distal to shoulder | 0 | 0 |
| Distal to occiput | 0 | 0 |
| Radiating to upper limb | 0 | 0 |
| Morning stiffness | 503 | 53.40 |
| Nocturnal pain | 375 | 39.81 |
| Pain intensity | 13 | 1.38 |
| Personal care | 13 | 1.38 |
| Lifting heavy objects | 13 | 1.38 |
| Reading | 13 | 1.38 |
| Headache | 13 | 1.38 |
| Concentration | 13 | 1.38 |
| Work | 13 | 1.38 |
| Sleep | 13 | 1.38 |
| Driving | 13 | 1.38 |
| Recreational activities | 13 | 1.38 |
| NDI | 13 | 1.38 |
| NRS | 0 | 0 |
| Flexion | 0 | 0 |
| Extension | 0 | 0 |
| Lateral Flexion | 0 | 0 |
| Rotation | 0 | 0 |
| Forward head posture | 122 | 12.95 |
| Shoulder protraction | 80 | 8.49 |
| Shoulders not level | 146 | 15.50 |
| Thoracic spine kyphosis | 48 | 5.10 |
| DCFET | 56 | 5.94 |
| Spurling test | 0 | 0 |
| Spring test-pain | 20 | 2.12 |
| Spring test-Hypomobility | 20 | 2.12 |
| Spring test-Hypermobility | 20 | 2.12 |
| Muscle tightness | 18 | 1.91 |
| Exacerbation on Flexion | 0 | 0 |
| Exacerbation on Extension | 0 | 0 |
| Exacerbation on Lateral Flexion | 0 | 0 |
| Exacerbation on Rotation | 0 | 0 |
| Prediction | 0 | 0 |

**Table S3 Comparison of Characteristics among the Training, Internal validation, and External Validation Sets**

|  | **Train Set (n=436)** | **Internal Validation Set (n=187)** | **External Validation Set (n=319)** | **p** |
| --- | --- | --- | --- | --- |
| **Age (mean (SD))** | 41.94 (9.97) | 42.99 (10.47) | 43.26 (14.17) | 0.255 |
| **Gender (%)** |  |  |  | <0.001* |
| Male | 138 (31.7) | 73 (39.2) | 151 (47.3) |  |
| Female | 298 (68.3) | 114 (60.8) | 168 (52.7) |  |
| **BMI (mean (SD))** | 23.87 (1.93) | 23.67 (1.66) | 23.65 (1.90) | 0.229 |
| **Symptom duration (mean (SD))** | 39.92 (53.03) | 36.77 (44.02) | 61.22 (78.87) | <0.001* |
| **Prior history of NP (%)** |  |  |  | 0.496 |
| Yes | 218 (50.0) | 85 (45.6) | 148 (46.4) |  |
| No | 218 (50.0) | 102 (54.4) | 171 (53.6) |  |
| **Pain laterality (%)** |  |  |  | 0.560 |
| Yes | 215 (49.0) | 87 (46.4) | 165 (51.7) |  |
| No | 222 (51.0) | 100 (53.6) | 154 (48.3) |  |
| **Distal to upper back (%)** |  |  |  | 0.593 |
| Yes | 221 (50.8) | 99 (52.8) | 153 (48.0) |  |
| No | 215 (49.2) | 88 (47.2) | 166 (52.0) |  |
| **Distal to shoulder (%)** |  |  |  | 0.510 |
| Yes | 217 (49.8) | 93 (49.6) | 146 (45.8) |  |
| No | 219 (50.2) | 94 (50.4) | 173 (54.2) |  |
| **Distal to occiput (%)** |  |  |  | 0.446 |
| Yes | 128 (29.3) | 54 (28.8) | 106 (33.2) |  |
| No | 308 (70.7) | 133 (71.2) | 213 (66.8) |  |
| **Radiating pain to upper limb (%)** |  |  |  | <0.001* |
| Yes | 111 (25.5) | 51 (27.2) | 143 (44.8) |  |
| No | 325 (74.5) | 136 (72.8) | 176 (55.2) |  |
| **Pain intensity (mean (SD))** | 2.03 (1.09) | 2.10 (1.04) | 2.01 (1.05) | 0.720 |
| **Personal care (mean (SD))** | 1.83 (1.57) | 1.69 (1.45) | 2.37 (1.73) | <0.001* |
| **Lifting heavy objects (mean (SD))** | 1.68 (1.49) | 1.77 (1.34) | 2.53 (1.65) | <0.001* |
| **Reading (mean (SD))** | 1.94 (1.23) | 1.81 (1.25) | 2.48 (1.69) | <0.001* |
| **Headache (mean (SD))** | 1.26 (1.58) | 1.22 (1.57) | 2.47 (1.65) | <0.001* |
| **Concentration (mean (SD))** | 1.76 (1.11) | 1.82 (1.16) | 2.67 (1.63) | <0.001* |
| **Work (mean (SD))** | 1.97 (1.14) | 2.05 (1.20) | 2.61 (1.69) | <0.001* |
| **Sleep (mean (SD))** | 1.87 (1.15) | 2.16 (1.24) | 2.46 (1.66) | <0.001* |
| **Driving (mean (SD))** | 1.94 (1.16) | 2.02 (1.22) | 2.36 (1.70) | <0.001* |
| **Recreational activities (mean (SD))** | 1.72 (1.50) | 1.67 (1.44) | 2.69 (1.65) | <0.001* |
| **NDI (mean (SD))** | 36.01 (8.05) | 36.62 (8.34) | 49.29 (9.89) | <0.001* |
| **NRS (mean (SD))** | 4.00 (1.83) | 4.11 (1.76) | 3.98 (1.81) | 0.783 |
| **Flexion (mean (SD))** | 38.62 (8.66) | 38.16 (8.42) | 35.95 (13.18) | 0.001* |
| **Extension (mean (SD))** | 33.63 (7.87) | 33.02 (7.76) | 31.76 (10.27) | 0.012* |
| **Lateral flexion (mean (SD))** | 32.01 (5.75) | 31.37 (5.50) | 31.27 (7.71) | 0.243 |
| **Rotation (mean (SD))** | 58.42 (9.64) | 57.06 (9.00) | 56.42 (12.78) | 0.030* |
| **Forward head posture (%)** |  |  |  | 0.514 |
| Yes | 56 (12.9) | 31 (16.8) | 43 (13.5) |  |
| No | 380 (87.1) | 156 (83.2) | 276 (86.5) |  |
| **Shoulder protraction (%)** |  |  |  | 0.147 |
| Yes | 72 (16.5) | 27 (14.4) | 67 (21.0) |  |
| No | 364 (83.5) | 160 (85.6) | 252 (79.0) |  |
| **Shoulders not level (%)** |  |  |  | 0.370 |
| Yes | 48 (11.0) | 13 (7.2) | 29 (9.1) |  |
| No | 388 (89.0) | 175 (92.8) | 290 (90.9) |  |
| **Thoracic spine kyphosis (%)** |  |  |  | 0.035* |
| Yes | 109 (24.9) | 40 (21.6) | 55 (17.2) |  |
| No | 327 (75.1) | 147 (78.4) | 264 (82.8) |  |
| **DCFET (mean (SD))** | 20.15 (8.13) | 20.89 (7.43) | 20.89 (7.43) | 0.235 |
| **Spurling test (%)** |  |  |  | <0.001* |
| Yes | 75 (17.1) | 27 (14.4) | 98 (30.7) |  |
| No | 361 (82.9) | 160 (85.6) | 221 (69.3) |  |
| **Spring test pain (%)** |  |  |  | <0.001* |
| Normal | 59 (13.5) | 25 (13.6) | 83 (26.0) |  |
| Single segment tenderness | 136 (31.1) | 76 (40.8) | 83 (26.0) |  |
| 2-3 segments tenderness | 199 (45.8) | 73(39.2) | 73 (22.9) |  |
| Extensive tenderness | 12 (9.6) | 12 (6.4) | 80 (25.1) |  |
| **Spring test hypomobility (%)** |  |  |  | 0.692 |
| Normal | 81 (18.5) | 45 (24.0) | 66 (20.7) |  |
| Single segment tenderness | 141 (32.3) | 55 (29.6) | 95 (29.8) |  |
| 2-3 segments tenderness | 128 (29.3) | 48 (25.6) | 101 (31.7) |  |
| Extensive tenderness | 87 (19.9) | 39 (20.8) | 57 (17.9) |  |
| **Spring test hypermobility (%)** |  |  |  | 0.625 |
| Normal | 230 (52.8) | 99 (52.8) | 159 (49.8) |  |
| Single segment tenderness | 82 (18.9) | 39 (20.8) | 79 (24.8) |  |
| 2-3 segments tenderness | 68 (15.5) | 28 (15.2) | 43 (13.5) |  |
| Extensive tenderness | 56 (12.9) | 21 (11.2) | 38 (11.9) |  |
| **Presence of muscle tightness (%)** |  |  |  | 0.002* |
| Normal | 73 (16.7) | 31 (16.8) | 89 (27.9) |  |
| Single muscle tension | 114 (26.1) | 42 (22.4) | 78 (24.5) |  |
| 2-3 muscles or muscle groups tension | 154 (35.3) | 73 (39.2) | 82 (25.7) |  |
| Extensive muscle tension | 95 (21.9) | 40 (21.6) | 70 (21.9) |  |
| **Exacerbation on flexion (%)** |  |  |  | 0.001* |
| Yes | 255 (58.4) | 111 (59.2) | 146 (45.8) |  |
| No | 181 (41.6) | 76 (40.8) | 173 (54.2) |  |
| **Exacerbation on extension (%)** |  |  |  | 0.564 |
| Yes | 229 (52.4) | 94 (50.4) | 155 (48.6) |  |
| No | 207 (47.6) | 93 (49.6) | 164 (51.4) |  |
| **Exacerbation on lateral flexion (%)** |  |  |  | 0.318 |
| Yes | 236 (54.2) | 100 (53.6) | 156 (48.9) |  |
| No | 200 (45.8) | 87 (46.4) | 163 (51.1) |  |
| **Exacerbation on rotation (%)** |  |  |  | 0.014* |
| Yes | 201 (46.0) | 112 (60.0) | 165 (51.7) |  |
| No | 235 (54.0) | 75 (40.0) | 154 (48.3) |  |
| **Benefit (%)** |  |  |  | 0.174 |
| High | 235 (53.8) | 100 (53.6) | 151 (47.3) |  |
| Low | 201 (46.2) | 87 (46.4) | 168 (52.7) |  |

**Table S4 Sensitivity Analysis of δ Adjustments for Numerical Variables in Low-Benefit and High-Benefit Groups**

| **Analysis** | **Features** | **Low-benefit (n=228)** | **High-benefit (n=335)** | ***P* value** |
| --- | --- | --- | --- | --- |
| Delta-adjusting: 25% | | | | |
| δ = 5.96 | BMI (mean (SD)) | 24.43 (2.52) | 24.27 (2.57) | 0.441 |
| δ = 9.82 | Symptom duration (mean (SD)) | 58.70 (64.60) | 22.96 (27.19) | <0.001* |
| δ = 0.51 | Pain intensity (mean (SD)) | 2.00 (1.05) | 2.10 (1.11) | 0.234 |
| δ = 0.45 | Personal care (mean (SD)) | 1.87 (1.57) | 1.76 (1.54) | 0.361 |
| δ = 0.43 | Lifting heavy objects (mean (SD)) | 1.73 (1.51) | 1.69 (1.42) | 0.767 |
| δ = 0.48 | Reading (mean (SD)) | 1.99 (1.26) | 1.86 (1.20) | 0.175 |
| δ = 0.31 | Headache (mean (SD)) | 1.31 (1.61) | 1.22 (1.55) | 0.46 |
| δ = 0.44 | Concentration (mean (SD)) | 1.72 (1.10) | 1.82 (1.14) | 0.289 |
| δ = 0.50 | Work (mean (SD)) | 2.05 (1.11) | 1.94 (1.19) | 0.256 |
| δ = 0.48 | Sleep (mean (SD)) | 1.96 (1.17) | 1.91 (1.18) | 0.559 |
| δ = 0.49 | Driving (mean (SD)) | 1.93 (1.14) | 2.00 (1.20) | 0.513 |
| δ = 0.43 | Recreational activities (mean (SD)) | 1.70 (1.47) | 1.72 (1.50) | 0.852 |
| δ = 9.03 | NDI (mean (SD)) | 36.52 (8.04) | 36.01 (8.31) | 0.441 |
| δ = 5.13 | DCFET (mean (SD)) | 20.48 (8.21) | 21.15 (7.32) | 0.283 |
| Delta-adjusting: 50% | | | | |
| δ = 11.91 | BMI (mean (SD)) | 25.03 (4.01) | 24.71 (3.77) | 0.315 |
| δ = 19.65 | Symptom duration (mean (SD)) | 58.90 (64.60) | 23.14 (27.49) | <0.001* |
| δ = 1.02 | Pain intensity (mean (SD)) | 2.00 (1.05) | 2.11 (1.11) | 0.225 |
| δ = 0.90 | Personal care (mean (SD)) | 1.87 (1.57) | 1.76 (1.54) | 0.371 |
| δ = 0.85 | Lifting heavy objects (mean (SD)) | 1.73 (1.51) | 1.70 (1.42) | 0.78 |
| δ = 0.96 | Reading (mean (SD)) | 2.00 (1.26) | 1.87 (1.21) | 0.183 |
| δ = 0.63 | Headache (mean (SD)) | 1.31 (1.61) | 1.22 (1.55) | 0.467 |
| δ = 0.88 | Concentration (mean (SD)) | 1.73 (1.11) | 1.83 (1.15) | 0.281 |
| δ = 0.99 | Work (mean (SD)) | 2.05 (1.12) | 1.95 (1.19) | 0.268 |
| δ = 0.96 | Sleep (mean (SD)) | 1.97 (1.17) | 1.91 (1.18) | 0.576 |
| δ = 0.98 | Driving (mean (SD)) | 1.94 (1.15) | 2.00 (1.21) | 0.5 |
| δ = 0.85 | Recreational activities (mean (SD)) | 1.71 (1.48) | 1.73 (1.50) | 0.84 |
| δ = 18.07 | NDI (mean (SD)) | 36.61 (8.25) | 36.15 (8.51) | 0.489 |
| δ = 10.27 | DCFET (mean (SD)) | 20.75 (8.45) | 21.47 (7.60) | 0.261 |
| Delta-adjusting: 100% | | | | |
| δ = 23.83 | BMI (mean (SD)) | 26.23 (7.40) | 25.60 (6.65) | 0.268 |
| δ = 39.29 | Symptom duration (mean (SD)) | 59.31 (64.69) | 23.49 (28.25) | <0.001* |
| δ = 2.04 | Pain intensity (mean (SD)) | 2.01 (1.07) | 2.12 (1.12) | 0.212 |
| δ = 1.80 | Personal care (mean (SD)) | 1.88 (1.58) | 1.78 (1.56) | 0.393 |
| δ = 1.70 | Lifting heavy objects (mean (SD)) | 1.74 (1.52) | 1.71 (1.42) | 0.806 |
| δ = 1.92 | Reading (mean (SD)) | 2.01 (1.27) | 1.88 (1.22) | 0.203 |
| δ = 1.26 | Headache (mean (SD)) | 1.32 (1.61) | 1.23 (1.56) | 0.482 |
| δ = 1.77 | Concentration (mean (SD)) | 1.74 (1.12) | 1.84 (1.16) | 0.269 |
| δ = 1.98 | Work (mean (SD)) | 2.06 (1.13) | 1.96 (1.21) | 0.296 |
| δ = 1.93 | Sleep (mean (SD)) | 1.98 (1.18) | 1.93 (1.20) | 0.612 |
| δ = 1.96 | Driving (mean (SD)) | 1.95 (1.16) | 2.02 (1.23) | 0.477 |
| δ = 1.71 | Recreational activities (mean (SD)) | 1.72 (1.49) | 1.74 (1.51) | 0.816 |
| δ = 36.13 | NDI (mean (SD)) | 36.80 (8.94) | 36.42 (9.30) | 0.6 |
| δ = 20.54 | DCFET (mean (SD)) | 21.28 (9.34) | 22.11 (8.69) | 0.25 |

**Table S5 Sensitivity Analysis of δ Adjustments for Categorical Variables in Low-Benefit and High-Benefit Groups**

| **Features** | **Low-benefit (n=288)** | **High-benefit (n=335)** | ***P* value** |
| --- | --- | --- | --- |
| **Delta-adjusting: 1** | | | |
| **Prior history of NP (%)** |  |  | 0.014* |
| Yes | 161 (55.9) | 153 (45.7) |  |
| No | 127 (44.1) | 183 (54.3) |  |
| **Forward head posture (%)** |  |  | 0.12 |
| Yes | 81 (28.1) | 75 (22.4) |  |
| No | 207 (71.9) | 260 (77.6) |  |
| **Shoulder protraction (%)** |  |  | 0.669 |
| Yes | 62 (21.5) | 78 (23.3) |  |
| No | 226 (78.5) | 257 (76.7) |  |
| **Shoulders not level (%)** |  |  | 0.851 |
| Yes | 66 (22.9) | 80 (23.9) |  |
| No | 222 (77.1) | 255 (76.1) |  |
| **Thoracic spine kyphosis (%)** |  |  | 0.73 |
| Yes | 81 (28.1) | 89 (26.6) |  |
| No | 207 (61.9) | 246 (73.4) |  |
| **Spring test pain (%)** |  |  | <0.001* |
| Normal | 53 (18.4) | 30 ( 9.0) |  |
| Single segment tenderness | 98 (34.0) | 105 (31.3) |  |
| 2-3 segments tenderness | 122 (42.4) | 151 (45.1) |  |
| Extensive tenderness | 15 ( 5.2) | 49 (14.6) |  |
| **Spring test hypomobility (%)** |  |  | 0.961 |
| Normal | 57 (19.8) | 61 (18.2) |  |
| Single segment tenderness | 92 (31.9) | 107 (31.9) |  |
| 2-3 segments tenderness | 80 (27.8) | 97 (29.0) |  |
| Extensive tenderness | 59 (20.5) | 70 (20.9) |  |
| **Spring test hypermobility (%)** |  |  | 0.053 |
| Normal | 149 (51.7) | 174 (51.9) |  |
| Single segment tenderness | 60 (20.8) | 65 (19.4) |  |
| 2-3 segments tenderness | 33 (11.5) | 60 (17.9) |  |
| Extensive tenderness | 46 (16.0) | 36 (10.7) |  |
| **Muscle tightness (%)** |  |  | <0.001* |
| Normal | 71 (24.7) | 32 ( 9.6) |  |
| Single muscle tension | 86 (29.9) | 71 (21.2) |  |
| 2-3 muscles or muscle groups tension | 94 (32.6) | 131 (39.1) |  |
| Extensive muscle tension | 37 (12.8) | 101 (30.1) |  |
| **Delta-adjusting: -1** | | | |
| **Prior history of NP (%)** |  |  | 0.006* |
| Yes | 155 (53.8) | 142 (42.4) |  |
| No | 133 (46.2) | 193 (57.6) |  |
| **Forward head posture (%)** |  |  | 0.235 |
| Yes | 39 (13.5) | 34 (10.1) |  |
| No | 249 (86.5) | 301 (89.9) |  |
| **Shoulder protraction (%)** |  |  | 0.646 |
| Yes | 40 (13.9) | 52 (15.5) |  |
| No | 248 (86.1) | 283 (84.5) |  |
| **Shoulders not level (%)** |  |  | 0.878 |
| Yes | 26 (9.0) | 28 (8.4) |  |
| No | 262 (91.0) | 307 (91.6) |  |
| **Thoracic spine kyphosis (%)** |  |  | 0.407 |
| Yes | 70 (24.3) | 71 (21.2) |  |
| No | 218 (75.7) | 264 (78.8) |  |
| **Spring test pain (%)** |  |  | <0.001* |
| Normal | 54 (18.8) | 34 (10.1) |  |
| Single segment tenderness | 102 (35.4) | 108 (32.2) |  |
| 2-3 segments tenderness | 123 (42.7) | 148 (44.2) |  |
| Extensive tenderness | 9 (3.1) | 45 (13.4) |  |
| **Spring test hypomobility (%)** |  |  | 0.955 |
| Normal | 60 (20.8) | 65 (19.4) |  |
| Single segment tenderness | 93 (32.3) | 106 (31.6) |  |
| 2-3 segments tenderness | 80 (27.8) | 98 (29.3) |  |
| Extensive tenderness | 55 (19.1) | 66 (19.7) |  |
| **Spring test hypermobility (%)** |  |  | 0.068 |
| Normal | 152 (52.8) | 178 (53.1) |  |
| Single segment tenderness | 59 (20.5) | 64 (19.1) |  |
| 2-3 segments tenderness | 35 (12.2) | 61 (18.2) |  |
| Extensive tenderness | 42 (14.6) | 32 (9.6) |  |
| **Muscle tightness (%)** |  |  | <0.001* |
| Normal | 72 (25.0) | 34 (10.1) |  |
| Single muscle tension | 87 (30.2) | 71 (21.2) |  |
| 2-3 muscles or muscle groups tension | 94 (32.6) | 134 (40.0) |  |
| Extensive muscle tension | 35 (12.2) | 96 (28.7) |  |

**Table S6 Model Performance Comparison for Different Feature Sets**

| **Feature Set** | **Model Name** | **AUC** | **Accuracy** | **F1 Score** | **Sensitivity** | **Specificity** | **PPV** | **NPV** |
| --- | --- | --- | --- | --- | --- | --- | --- | --- |
| **Union** | MLP | 0.823 | 0.759 | 0.757 | 0.842 | 0.663 | 0.746 | 0.781 |
|  | RF | 0.806 | 0.754 | 0.753 | 0.802 | 0.698 | 0.757 | 0.75 |
|  | KNN | 0.805 | 0.733 | 0.731 | 0.792 | 0.663 | 0.734 | 0.731 |
|  | LGBM | 0.804 | 0.765 | 0.764 | 0.792 | 0.733 | 0.777 | 0.75 |
|  | GNB | 0.801 | 0.711 | 0.699 | 0.881 | 0.512 | 0.679 | 0.786 |
|  | XGB | 0.792 | 0.749 | 0.747 | 0.812 | 0.674 | 0.745 | 0.753 |
|  | Bagging | 0.785 | 0.695 | 0.692 | 0.782 | 0.593 | 0.693 | 0.699 |
|  | AdaBoost | 0.773 | 0.722 | 0.715 | 0.851 | 0.57 | 0.699 | 0.766 |
|  | DT | 0.744 | 0.636 | 0.628 | 0.772 | 0.477 | 0.634 | 0.641 |
| **Boruta** | LGBM | 0.777 | 0.695 | 0.69 | 0.802 | 0.57 | 0.686 | 0.71 |
|  | RF | 0.774 | 0.695 | 0.69 | 0.802 | 0.57 | 0.686 | 0.71 |
|  | MLP | 0.759 | 0.695 | 0.687 | 0.832 | 0.535 | 0.677 | 0.73 |
|  | XGB | 0.757 | 0.679 | 0.671 | 0.812 | 0.523 | 0.667 | 0.703 |
|  | DT | 0.739 | 0.679 | 0.677 | 0.752 | 0.593 | 0.685 | 0.671 |
|  | Bagging | 0.737 | 0.647 | 0.637 | 0.792 | 0.477 | 0.64 | 0.661 |
|  | KNN | 0.734 | 0.679 | 0.67 | 0.822 | 0.512 | 0.664 | 0.71 |
|  | GNB | 0.731 | 0.631 | 0.594 | 0.901 | 0.314 | 0.607 | 0.73 |
|  | AdaBoost | 0.708 | 0.674 | 0.665 | 0.812 | 0.512 | 0.661 | 0.698 |
| **Lasso** | LGBM | 0.8 | 0.743 | 0.742 | 0.792 | 0.686 | 0.748 | 0.738 |
|  | RF | 0.795 | 0.738 | 0.736 | 0.802 | 0.663 | 0.736 | 0.74 |
|  | MLP | 0.795 | 0.738 | 0.737 | 0.792 | 0.674 | 0.741 | 0.734 |
|  | GNB | 0.785 | 0.701 | 0.687 | 0.881 | 0.488 | 0.669 | 0.778 |
|  | XGB | 0.785 | 0.738 | 0.736 | 0.802 | 0.663 | 0.736 | 0.74 |
|  | Bagging | 0.784 | 0.706 | 0.703 | 0.782 | 0.616 | 0.705 | 0.707 |
|  | KNN | 0.783 | 0.706 | 0.705 | 0.752 | 0.651 | 0.717 | 0.691 |
|  | AdaBoost | 0.768 | 0.711 | 0.703 | 0.851 | 0.547 | 0.688 | 0.758 |
|  | DT | 0.765 | 0.717 | 0.715 | 0.772 | 0.651 | 0.722 | 0.709 |
| **Intersection** | RF | 0.743 | 0.684 | 0.677 | 0.812 | 0.535 | 0.672 | 0.708 |
|  | LGBM | 0.739 | 0.695 | 0.691 | 0.792 | 0.581 | 0.69 | 0.704 |
|  | AdaBoost | 0.732 | 0.668 | 0.658 | 0.822 | 0.488 | 0.654 | 0.7 |
|  | XGB | 0.732 | 0.684 | 0.678 | 0.802 | 0.547 | 0.675 | 0.701 |
|  | MLP | 0.727 | 0.663 | 0.654 | 0.802 | 0.5 | 0.653 | 0.683 |
|  | DT | 0.716 | 0.684 | 0.683 | 0.752 | 0.605 | 0.691 | 0.675 |
|  | Bagging | 0.711 | 0.658 | 0.651 | 0.782 | 0.512 | 0.653 | 0.667 |
|  | GNB | 0.7 | 0.604 | 0.562 | 0.881 | 0.279 | 0.589 | 0.667 |
|  | KNN | 0.691 | 0.62 | 0.606 | 0.792 | 0.419 | 0.615 | 0.632 |

**Table S7. Optimal Hyperparameters for 9 Machine Learning Models**

| **Model** | **Parameter** | **Value** |
| --- | --- | --- |
| MLP Classifier | hidden_layer_sizes | (50, 50) |
|  | max_iter | 1000 |
|  | activation | tanh |
|  | solver | sgd |
|  | alpha | 0.0001 |
|  | random_state | 42 |
| K-Nearest Neighbor | n_neighbors | 15 |
|  | weights | distance |
|  | metric | manhattan |
| Gaussian Naïve Bayesian | - | Default parameters |
| Decision Tree | max_depth | 5 |
|  | min_samples_split | 10 |
|  | min_samples_leaf | 5 |
|  | random_state | 42 |
| Random Forest | n_estimators | 100 |
|  | max_depth | 10 |
|  | min_samples_split | 10 |
|  | min_samples_leaf | 10 |
|  | random_state | 42 |
| AdaBoost Classifier | n_estimators | 50 |
|  | learning_rate | 0.1 |
|  | random_state | 42 |
| Bagging Classifier | base_estimator | Decision Tree (max_depth=5, minsamples_split=10) |
|  | n_estimators | 100 |
|  | random_state | 42 |
| Extreme Gradient Boosting | max_depth | 3 |
|  | n_estimators | 200 |
|  | learning_rate | 0.01 |
|  | colsample_bytree | 0.8 |
|  | subsample | 0.8 |
|  | random_state | 42 |
| Light Gradient Boosting Machine | num_leaves | 31 |
|  | learning_rate | 0.01 |
|  | max_depth | 7 |
|  | n_estimators | 200 |
|  | min_child_weight | 1.0 |
|  | random_state | 42 |


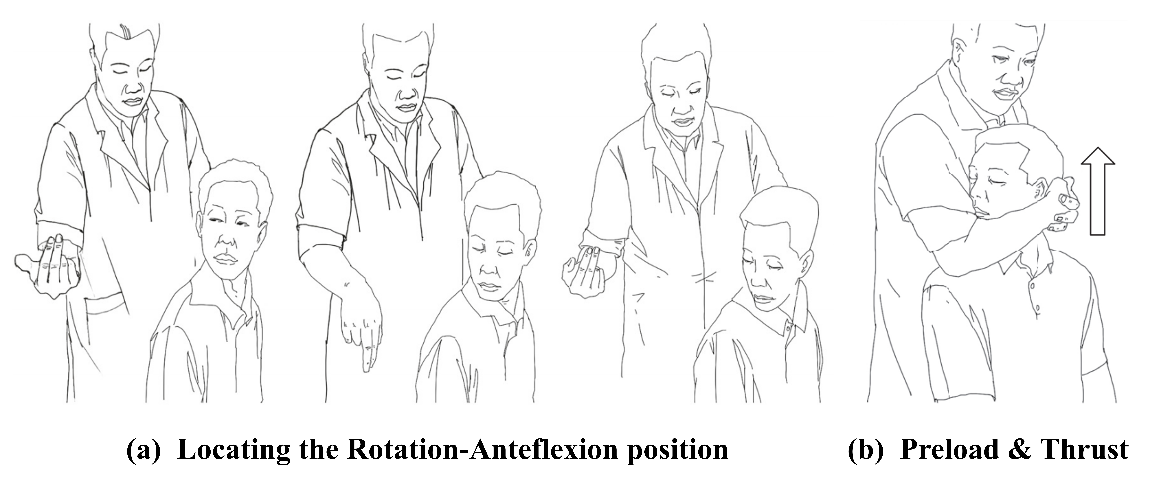


**Figure S1 Procedure of standardized SMT procedure.** (A) shows the subject’s active rotary-position process of rotation-flexion-rotation under guidance. (B) shows a physiotherapist performing preload traction and upward-thrust.





**Figure S2: Density Plots of Imputed Data for Numerical and Categorical Variables.** BMI: Body Mass Index; DCFET: Deep Cervical Flexor Endurance Test; NDI: Neck disability index.


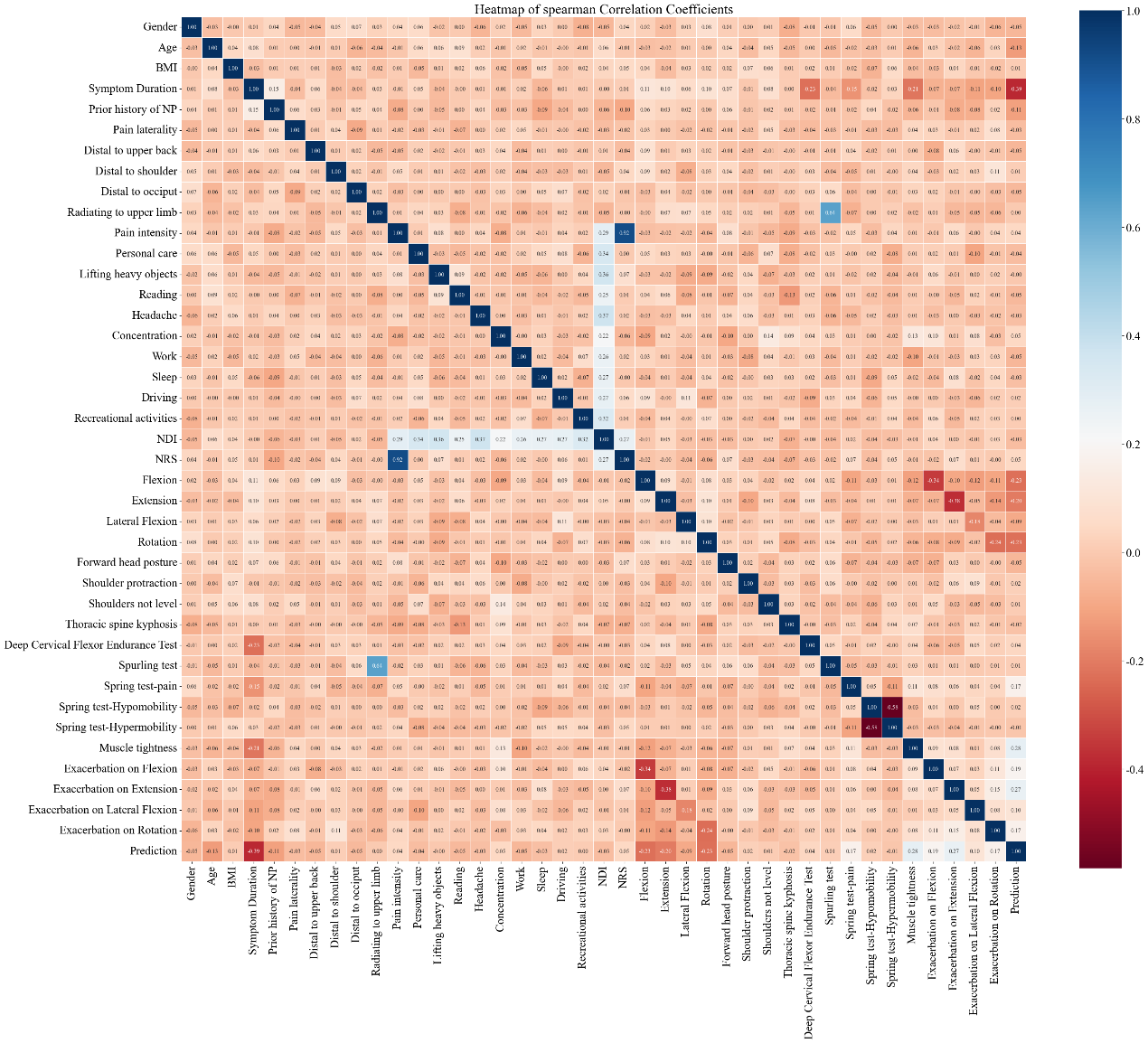


**Figure S3 Heatmap of Spearman Correlation Coefficients of All Features**


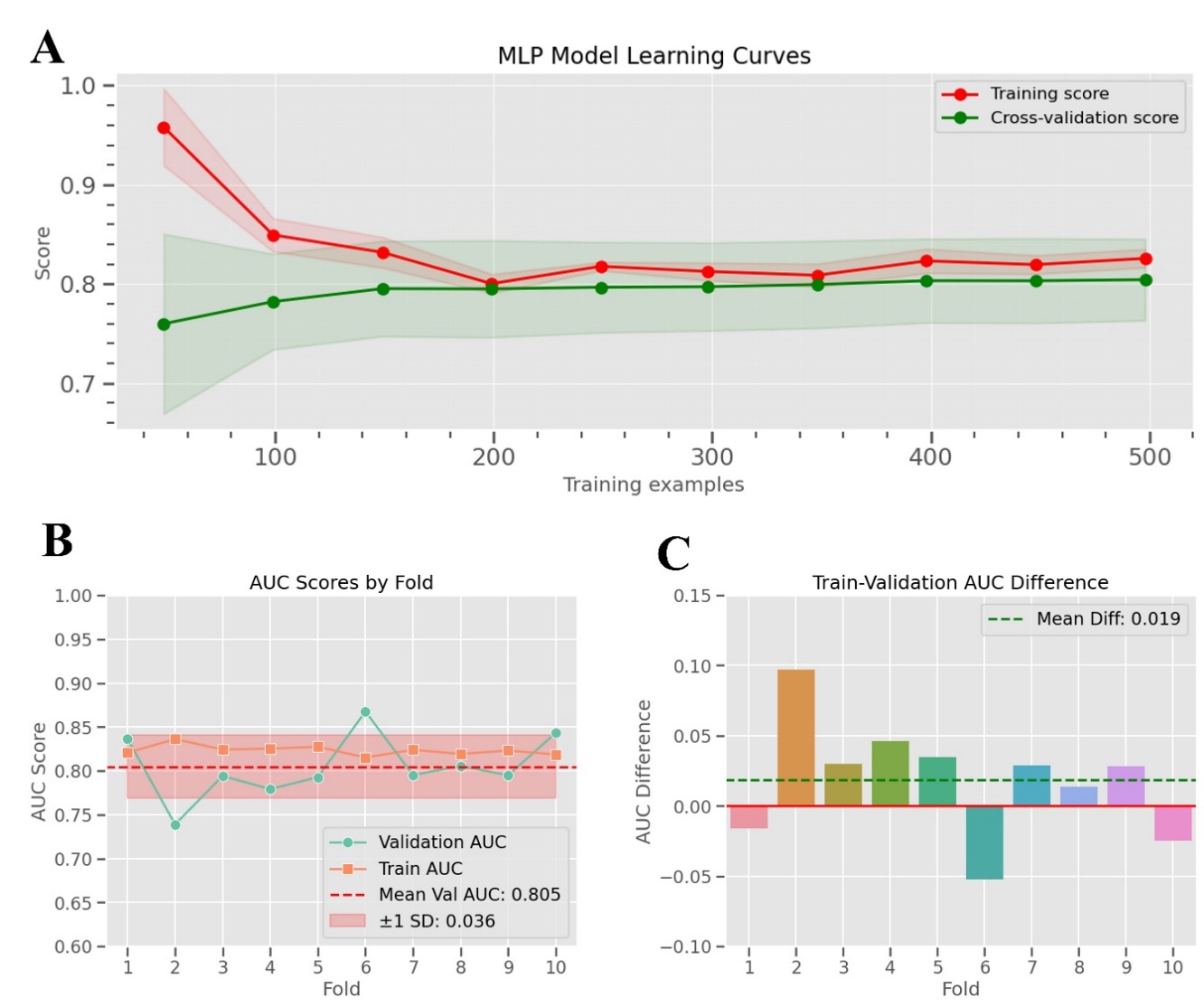


**Figure S4. Analysis of MLP Model Robustness and Overfitting Prevention.** A: MLP model learning curves showing AUC score changes for training set (red line) and cross-validation set (green line) across different training sample sizes. B: Comparison of AUC scores in 10-fold cross-validation. Green line represents validation AUC for each fold, orange line shows training AUC for each fold, and red dashed line indicates mean validation AUC. C: Bar chart showing differences between training and validation AUC scores, with green dashed line indicating the average difference.


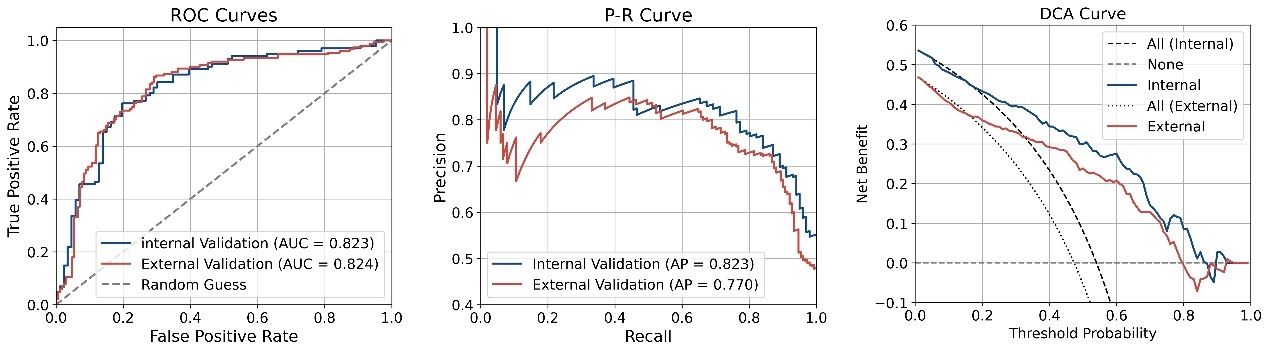


**Figure S5. Performance evaluation of the model in external validation.** (A) ROC curves comparing the internal and external validation sets. (B) P-R curves showing the precision and recall performance of the model in both internal and external validation. (C) DCA curves assessing the net benefit of the model at different threshold probabilities for internal and external validations.


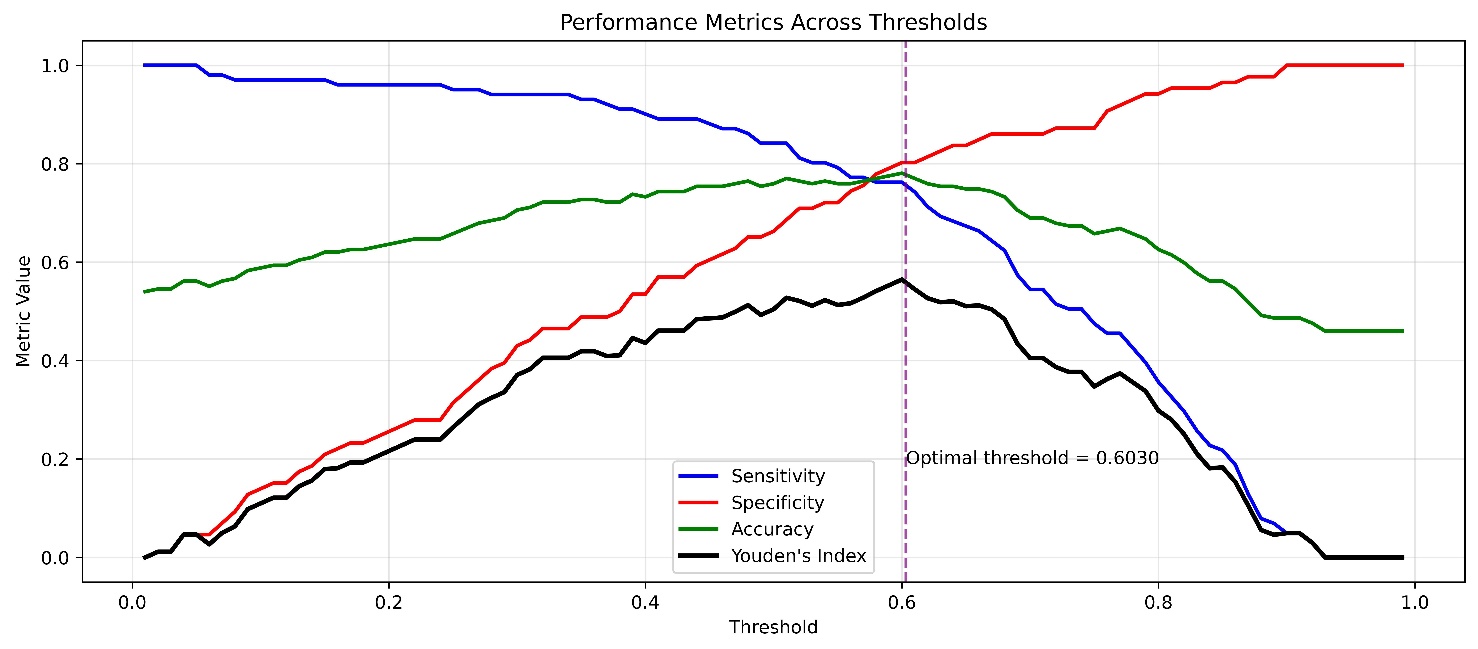


**Figure S6: Performance metrics across probability thresholds for the MLP model.** Sensitivity (blue), specificity (red), accuracy (green), and Youden's Index (black). The vertical dashed line indicates the optimal threshold of 0.6030, where Youden's Index reaches its maximum value of 0.565, yielding balanced performance metrics (sensitivity=0.762, specificity=0.802, accuracy=0.781, PPV=0.819, NPV=0.742).


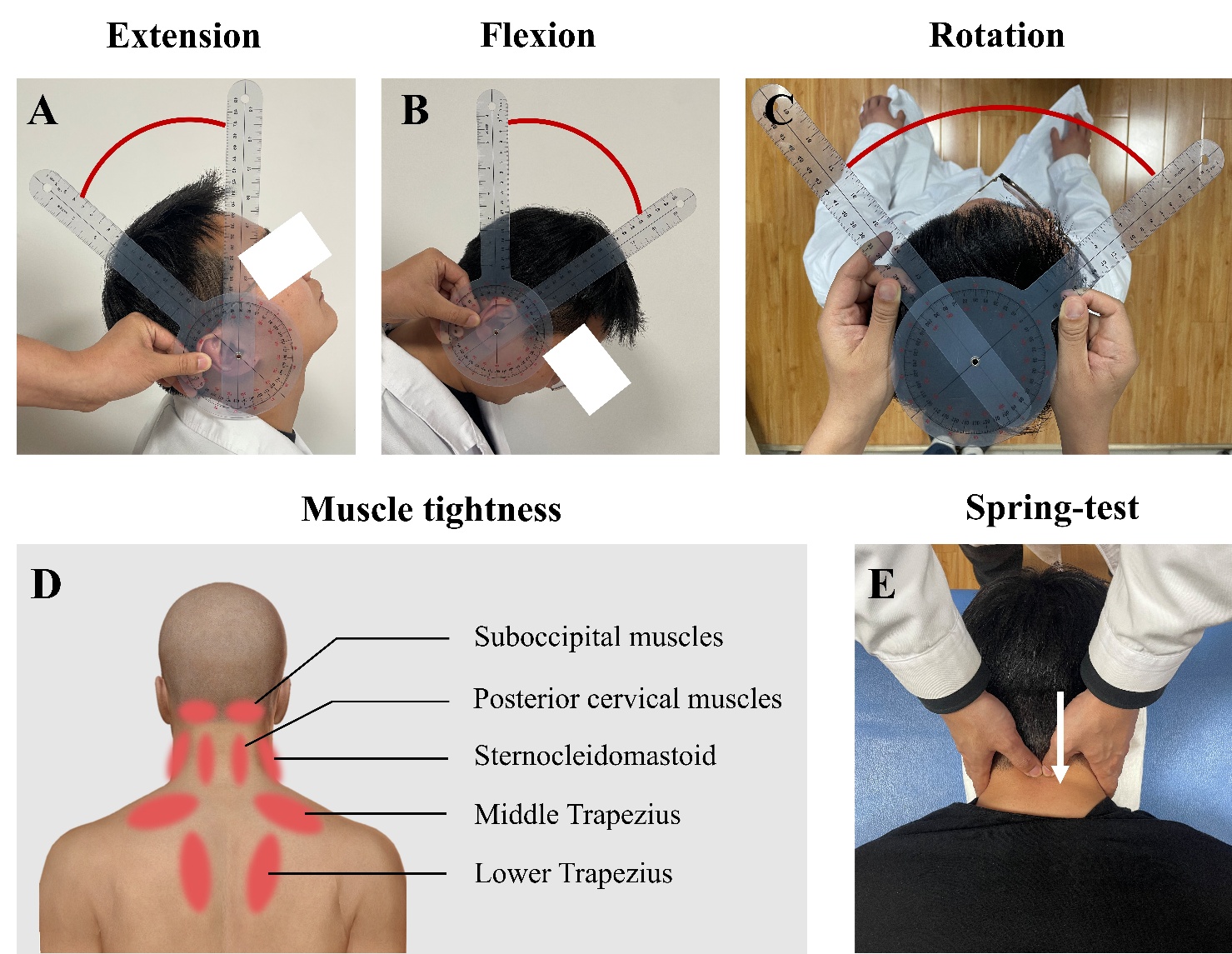


**Figure S7: Clinical Assessment Methods for Key Predictors.** Panels A-C illustrate the measurement techniques for cervical range of motion: extension (A), flexion (B), and rotation (C). Measurements were performed using a standard universal goniometer with the clinician positioning one arm of the goniometer vertically while aligning the other arm with the subject's head movement. For flexion and extension (A-B), the ear canal served as the axis of rotation, with the angle between the starting and maximum positions recorded. For rotation (C), the vertex of the head served as the axis, with the goniometer arms aligned with the nose at maximum rotation in each direction to determine the total rotational range. Panel D depicts the assessment regions for muscle tightness evaluation, which was performed through manual palpation of cervical musculature at 10 sites (5 per side). Findings were categorized as: normal (no tension), mild (single site involvement), moderate (2-3 sites), or extensive (>3 sites). Panel E demonstrates the spring test pain procedure, performed with the patient in prone position, where the examiner applied posteroanterior pressure to each cervical spinous process. Patient-reported pain responses were categorized as: normal (no pain), mild (single segment pain), moderate (2-3 segments), or widespread (>3 segments).
